# siRNA screen of ES cell-derived motor neurons identifies novel regulators of tetanus toxin and neurotrophin receptor trafficking

**DOI:** 10.3389/fncel.2014.00140

**Published:** 2014-05-20

**Authors:** Marco Terenzio, Matthew Golding, Giampietro Schiavo

**Affiliations:** ^1^Molecular NeuroPathobiology Laboratory, Cancer Research UK London Research InstituteLondon, UK; ^2^Sobell Department of Motor Neuroscience and Movement Disorders, UCL Institute of Neurology, University College LondonLondon, UK

**Keywords:** axonal transport, BICD1, embryonic stem cells, endocytosis, tetanus neurotoxin, p75^NTR^

## Abstract

Neurons rely on the long-range transport of several signaling molecules such as neurotrophins and their receptors, which are required for neuronal development, function and survival. However, the nature of the machinery controlling the trafficking of signaling endosomes containing activated neurotrophin receptors has not yet been completely elucidated. We aimed to identify new players involved in the dynamics of neurotrophin signaling endosomes using a medium-throughput unbiased siRNA screening approach to quantify the intracellular accumulation of two fluorescently tagged reporters: the binding fragment of tetanus neurotoxin (H_C_T), and an antibody directed against the neurotrophin receptor p75^NTR^. This screen performed in motor neurons differentiated from mouse embryonic stem (ES) cells identified a number of candidate genes encoding molecular motors and motor adaptor proteins involved in regulating the intracellular trafficking of these probes. Bicaudal D homolog 1 (BICD1), a molecular motor adaptor with pleiotropic roles in intracellular trafficking, was selected for further analyses, which revealed that BICD1 regulates the intracellular trafficking of H_C_T and neurotrophin receptors and likely plays an important role in nervous system development and function.

## Introduction

The development, maintenance, and function of the vertebrate nervous system rely on a complex network of signaling pathways tightly regulated in space and time by different growth factors. Amongst these, neurotrophins represent a very important class of molecules, which activate two different types of receptors, the Trk family of receptor tyrosine kinases and p75^NTR^, a member of the TNF receptor superfamily. Upon neurotrophin binding at the plasma membrane, Trk receptors are endocytosed and can then either signal locally, or be retrogradely transported along the axon to the neuronal cell body, where they initiate signaling cascades leading to the long-term modulation of gene expression (Heerssen and Segal, 2002).

Different types of neurons express distinct repertoires of Trk receptors, which determine their responsiveness and reliance on specific neurotrophins. In particular, motor neurons located in the ventral horn of the spinal cord express TrkB, TrkC, and p75^NTR^, and functionally connect skeletal muscles with the motor cortex via the corticospinal tract. The survival of motor neurons is ensured *in vivo* by neurotrophins secreted by their target muscles. Once bound to the motor neuron surface, neurotrophin receptor complexes are transported in axonal signaling endosomes, which ensure the long-range intracellular transport and somatic endocytic sorting of these receptors (Bronfman et al., 2007; Schmieg et al., 2013).

Interestingly, this route constitutes a major gateway for the entry and dissemination of pathogens in the nervous system. Specifically, motor neuron signaling endosomes mediate the axonal transport of neurotropic viruses, such as poliovirus and canine adenovirus 2 (Ohka et al., 2009; Salinas et al., 2009), and clostridial neurotoxins, such as tetanus toxin (TeNT) and botulinum neurotoxin (BoNT) (Restani et al., 2012; Bercsenyi et al., 2013).

TeNT is taken up at the neuromuscular junction (NMJ) and transported to the spinal cord, where it enters inhibitory interneurons and blocks neurotransmitter release (Salinas et al., 2010). The binding fragment of TeNT (H_C_T) has been shown to share the same route of internalization and axonal retrograde transport with neurotrophins and their receptors (Deinhardt et al., 2006), and it has the advantages of being non-toxic and readily expressed in a recombinant form with small peptide tags suitable for chemical and fluorescent labeling (Bohnert and Schiavo, 2005; Deinhardt et al., 2006). As such, H_C_T represents a reliable probe for the analysis of the axonal retrograde transport of signaling endosomes in motor neurons *in vitro* and *in vivo*.

RNAi screens have been widely used to generate complex datasets from image-based analysis of cell lines in high-throughput formats (Krausz, 2007; Demir and Boutros, 2012). However, highly proliferative cell lines do not constitute the most physiologically relevant model system to study postmitotic cells such as neurons. On the other hand, the use of primary motor neurons for high-content image-based RNAi screens presents daunting challenges because of the technical difficulties associated with generating enough cells, transfecting them efficiently and imaging their complex morphology in a quantitative and unbiased manner.

In spite of these obstacles, we developed a reliable protocol to transfect motor neurons with a library of short interference RNAs (siRNA) targeting a panel of molecular motors and their adaptors. This screen identified a small cohort of genes, which affected the intracellular accumulation of H_C_T and p75^NTR^ when silenced. Knockdown of one gene in particular, *bicaudal D homolog 1* (*Bicd1*) yielded an increased internalization phenotype for H_C_T and was selected for further analyses, which demonstrated that BICD1 depletion also increased the intracellular accumulation of ligand-bound p75^NTR^ and TrkB (Terenzio et al., 2014).

BICD1 is known to be involved in intracellular trafficking, dynein-mediated transport (Hoogenraad et al., 2001; Matanis et al., 2002; Bianco et al., 2010; Aguirre-Chen et al., 2011), and has important roles in the development and function of the *Drosophila* and *C. elegans* nervous systems (Li et al., 2010). We now show that BICD1 is a key regulator of H_C_T and neurotrophin receptor dynamics and performs this role by controlling the endocytic sorting of H_C_T and neurotrophin receptors to regulate their recycling to the plasma membrane. Our data identify a novel function for BICD1 as a modulator of axonal signaling endosome dynamics, which may also be relevant for different growth factor receptors and virulence factors in other cellular systems.

## Materials and methods

### Ethics statement

All experiments were carried out following the guidelines of the Cancer Research UK genetic manipulation procedures.

### Reagents and antibodies

All chemicals were supplied by Sigma, unless stated otherwise. Tissue culture media and supplements were from Life Technologies.

The βIII tubulin (TUJ1) antibody was from Covance and the Vamp2 antibody (clone 69.1) was from Synaptic Systems. The characterization of the polyclonal antibody against the extracellular domain of p75^NTR^ (αp75^NTR^; 5411) was previously described (Deinhardt et al., 2007). The Vps26 antibody was a kind gift of Matthew Seaman (University of Cambridge, UK).

### Motor neuron cultures

For the preparation of embryonic stem (ES) cell-derived motor neurons, ES cells were maintained on fish skin gelatin coated flasks in Glasgow Minimal Essential Medium (GMEM), 5% ES cell-qualified foetal bovine serum (FBS), 5% knockout serum replacement (KSR), 1% GLUTAMAX, 0.1 mM 2-mercaptoethanol and 1000 units/ml of leukaemia inhibitory factor (ESGRO, Millipore). To generate motor neurons, 1.5 × 10^6^ ES cells were grown in suspension on a 10 cm non-tissue culture treated Petri dish containing differentiation (DFNK) medium: 45% Neurobasal, 45% DMEM/Ham's-F12, 10% KSR, 1% GLUTAMAX and 0.1 mM 2-mercaptoethanol.

The following day, EBs were gently centrifuged and re-suspended in 10 ml of fresh DFNK medium and plated on a new Petri dish. 24 h later, the greatly enlarged EBs were allowed to sediment by gravity and re-suspended in fresh DFNK medium supplemented with 1 μM all-trans retinoic acid (RA) and 333 nM Smoothened Agonist (SAG; Enzo Life Sciences). EBs were maintained under these conditions for a further 4 days with the medium changed every other day. EBs were then dissociated with 0.025% porcine pancreatic trypsin in 1 ml PBS for 7 min at 37°C and processed as described previously for the dissociation of mouse primary E13.5 spinal cord motor neurons (Hafezparast et al., 2003).

Cells were plated onto poly-D-ornithine and laminin coated dishes in motor neuron growth medium: neurobasal medium supplemented with 2% B27, 2% heat-inactivated horse serum, 1% GLUTAMAX, 25 μM 2-mercaptoethanol, 10 ng/ml rat ciliary neurotrophic factor (CNTF; R&D Systems), 100 pg/ml rat glial cell line-derived neurotrophic factor (GDNF; R&D Systems) and 1 μM RA.

### Generation of homozygous RRP227 ES cells

Mouse ES cells with a gene-trap insertion in the first intron of *Bicd1* (RRP227; http://www.informatics.jax.org/javawi2/servlet/WIFetch?page=alleleDetailandkey=544886) were obtained from the Mutant Mouse Regional Resource Center. Homozygous *Bicd1*^gt/gt^ cells were generated as previously described in Lefebvre et al. (2001) and Terenzio et al. (2014).

### Cell transfection

Full length mouse *Bicd1* cDNA (MGC-27566, LGC Promochem) was cloned into the EcoR1/BamH1 sites of the EGFP-N1 vector (Clontech) and microinjected into motor neurons as previously described (Deinhardt et al., 2006). BICD1-GFP expressing cells were imaged after 24 h using an inverted LSM510 confocal microscope (Zeiss). N2A cells were cultured and transfected as previously described (Terenzio et al., 2014).

### siRNA delivery protocol optimization

An individual mix was prepared for 23 different lipid-based transfection reagents (0.25 μ l of the reagent and 9.75 μ l OptiMEM per well). Ten micro liters of this mixture were added to 10 μ l VAMP2 siRNA diluted in OptiMEM. The transfection mix was then dispensed into each well of poly-D-ornithine/laminin coated 96-well glass bottomed plates (IWAKI) and incubated at room temperature for 20 min. For each well, 3.5 × 10^4^ HBG3 ES cell-derived motor neurons (Wichterle et al., 2002) suspended in 80 μ l of motor neuron growth medium were plated on top of the transfection mixture. After 72 h incubation, motor neurons we fixed in 4% paraformaldehyde (PFA) and immunostained for VAMP2 and βIII tubulin.

The Thermo Scientific™ ArrayScan™ XTI High Content Analysis Reader was used for automated image collection and image analysis was performed using the spot counting algorithm. To assess transfection-related toxicity, each transfection reagent was scored on the basis of the number of viable and morphologically intact motor neurons remaining after transfection. Based on favorable cell viability scores and effective knockdown of VAMP2 protein levels, eight transfection reagents were then selected for a second round of validation. Dreamfect Gold (OZ Biosciences) was chosen to conduct the siRNA screen as it proved to be the most effective in reliably inducing VAMP2 knock-down in both primary and ES cell-derived motor neurons in a 96-well format using the aforementioned reverse transfection protocol.

### siRNA screening

A master mix of Dreamfect Gold transfection reagent sufficient for the entire siRNA library targeting 133 genes was prepared (0.25 μ l Dreamfect Gold and 9.75 μ l OptiMEM per well) and 10 μ l added to each well of another 96-well plate containing 10 μ l siRNA (50 nM siRNA in OptiMEM) targeting a single gene (Supplementary Table 1A, B). Twenty micro liters of transfection mix were then dispensed into each well of poly-D-ornithine/laminin coated 96-well glass bottomed plates (IWAKI) and incubated at room temperature for 20 min to 1 h. For each well, 3.5 × 10^4^ HBG3 ES cell derived motor neurons suspended in 80 μ l of motor neuron growth medium were plated on top of the transfection mixture (50 nM final concentration for each siRNA). The cells were then left to grow for 72 h before performing the internalization assay.

To assess for probe internalization, the growth medium was removed and replaced with Neurobasal medium containing 20 nM AlexaFluor555-conjugated H_C_T and 0.4 μg/ml αp75^NTR^ (Deinhardt et al., 2007). Positive control wells previously incubated with RISC-free siRNA (Qiagen) were additionally supplemented with 1 mM EHNA. Motor neurons were then allowed to internalize the probes at 37°C for 2 h, cooled on ice for 5 min, acid-washed (100 mM citric acid pH 2.0, 142 mM NaCl) for 2 min, washed and fixed with 4% w/v PFA for 20 min. To detect internalized αp75^NTR^, cells were immunostained with anti-rabbit AlexaFluor647-conjugated secondary antibody. Finally, each plate was sealed and 16 confocal Z-stacks were acquired for each well using an inverted LSM510 confocal microscope (Zeiss). Internalized probes were quantified using Cell Profiler as described in the data quantification section.

### Transmission electron microscopy

H_C_T was conjugated to colloidal gold by mixing 250 μg of purified H_C_T in 0.5 ml of 2 mM sodium tetraborate to 1 ml of colloidal gold particles (10 nm; British Biocell) as described elsewhere Terenzio et al. (2014).

Motor neurons plated on coverslips were incubated with 20 nM nanogold-conjugated H_C_T for 2 h at 37°C prior to washing. Cells were fixed in 2.5% glutaraldehyde, 4% PFA in Sorensen's phosphate buffer at room temperature for 20 min, postfixed in osmium tetroxide, stained with tannic acid and dehydrated progressively up to 100% ethanol. Finally, coverslips were embedded in an Epon epoxy resin, sectioned (70–75 nm) and stained with lead citrate. Images were acquired using a Tecnai Spirit Biotwin (FEI) transmission electron microscope.

### Assessment of axonal retrograde transport

Axonal retrograde transport kinetics of H_C_T and αp75^NTR^ in wild type and in *Bicd1*^gt/gt^ motor neurons was performed as previously described (Lalli and Schiavo, 2002; Deinhardt et al., 2006) and quantified using Motion Analysis software (Kinetic Imaging) (Bohnert and Schiavo, 2005; Deinhardt et al., 2006).

### Immunofluorescence

Motor neurons were seeded onto poly-D-ornithine and laminin-coated coverslips and maintained under standard culture conditions for 4–5 days. Cells were then fixed with 4% PFA for 15 min at room temperature, washed and blocked with 2% bovine serum albumin (BSA) in PBS containing 0.2% Triton X-100 for 20 min at room temperature. Coverslips were then incubated for 1 h with primary antibodies followed by AlexaFluor-conjugated secondary antibodies (1:500; Life Technologies). Finally, samples were postfixed with PFA and mounted in Mowiol.

### Western blotting

SDS-PAGE was performed using 4–12% NuPAGE Bis-Tris gradient gels (Life Technologies) according to the manufacturer's instructions and blotted onto polyvinylidene fluoride (PVDF) membranes (Millipore). Membranes were blocked in 5% skimmed milk dissolved in Tris-buffered saline (TBS) containing 0.05% Tween-20 (TBST) for 1 h at room temperature and then incubated with primary antibodies diluted 1:1000 or 1:2000 in TBST for 1 h at room temperature or overnight at 4°C. Blots were then washed and incubated with appropriate HRP-conjugated secondary antibodies (GE Healthcare). Immunoreactivity was detected using Luminata or Crescendo ECL substrates (Millipore) and ECL-Hyperfilm (GE-Healthcare).

### Data quantification

Quantification of western blots and immunofluorescence staining was performed using ImageJ for all experiments except the siRNA screen and the validation re-screen. Typically, the fluorescence staining of the area of interest was thresholded and the number of thresholded voxels measured. Immunostaining for neuronal-specific proteins, such as βIII tubulin, was used for normalization purposes.

For the siRNA screen, image quantification of fluorescent H_C_T and αp75^NTR^ was performed using Cell Profiler (http://www.cellprofiler.org). RGB images were split and the green GFP channel (motor neuron reporter) was used to create a cell mask, which was then subtracted from the red AlexaFluor555-H_C_T and blue AlexaFluor647-αp75^NTR^ channels (Figure 2A). Individual H_C_T and p75^NTR^-positive organelles were then identified in each motor neuron and quantified.

## Results

### Validation of ES cell-derived motor neurons for high-throughput image analysis

A key requisite to successfully perform an siRNA screen is the ability to reliably and efficiently deliver RNA duplexes to the cytoplasm of target cells. However, motor neurons are notoriously difficult to transfect using conditions that do not affect their intracellular trafficking (Deinhardt and Schiavo, 2005). To overcome this technical challenge, we established a lipid-based reverse transfection method in primary motor neurons, which could be adapted to a 96-well plate format (see Material and Methods). To optimize the conditions required for efficient gene knockdown in preparation for the siRNA screen, we targeted VAMP2, since loss of this abundant SNARE protein does not affect neuron survival (Schoch et al., 2001). After testing 23 different transfection reagents, some of which are shown in Supplementary Figure 1, Dreamfect Gold (OZ Biosciences) was chosen on the basis of its ability to efficiently induce VAMP2 knockdown in primary motor neurons 3 days after transfection. Importantly, DreamFect Gold induced efficient depletion of VAMP2 without affecting cell viability or morphology (data not shown).

Because of the limited amount of material and the intrinsic cellular heterogeneity of embryo-derived primary motor neuron cultures, we resorted to using transgenic HB9-GFP (HBG3) ES cells to generate the large number of GFP-positive motor neurons necessary for our screen and was a prerequisite for the medium throughput imaging-based approach planned for this project.

**Figure 1:**
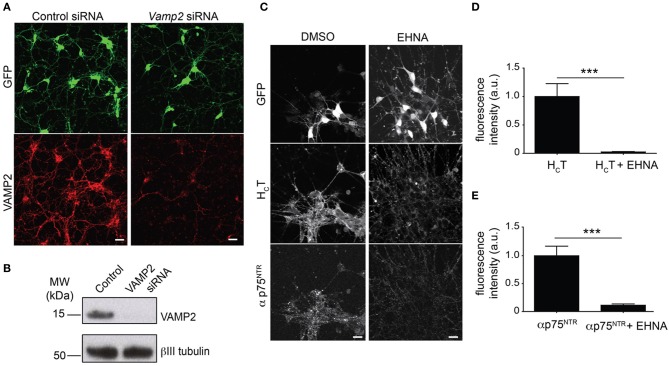
**Optimization of the read-out for the siRNA screen**. **(A)** Representative images of GFP-positive HBG3 ES cell-derived motor neurons (green) reverse transfected with *Vamp2* siRNA using Dreamfect Gold in a 96-well plate format and immunostained for VAMP2 (red). Scale bar, 20 μm. **(B)** Western blot analysis of VAMP2 after siRNA mediated *Vamp2* knock-down in HBG3 ES cell-derived motor neurons transfected in a 96-well format. **(C)** HBG3 ES cell-derived motor neurons expressing GFP (upper panels) were incubated with AlexaFluor555-conjugated H_C_T (middle panels) and αp75^NTR^ (lower panels) in the presence of 1 mM EHNA or DMSO vehicle control for 2 h at 37°C. Cells were then acid-washed, fixed and immunostained with AlexaFluor488-conjugated anti-rabbit IgG. The intracellular accumulation of both probes was substantially reduced by EHNA treatment, indicating that cytoplasmic dynein plays a major role in this process. Scale bar, 20 μm. **(D,E)** Quantification of internalized H_C_T **(D)** and αp75^NTR^ **(E)** in motor neurons exposed to H_C_T and αp75^NTR^ from three independent experiments with or without EHNA (paired *t*-test, mean ± s.e.m., ^***^p = 0.001).

HB9-GFP ES cells were readily expanded and differentiated into motor neurons with the expression of GFP driven by the Hb9 homeobox gene enhancer, which facilitates the unequivocal identification of motor neurons (Supplementary Figure 2) (Wichterle et al., 2002). We then confirmed that the transfection protocol previously optimized for primary cells in a 96-well format was also effective in efficiently knocking down VAMP2 in HB9-GFP ES cell-derived motor neurons in the same format (Figures 1A,B).

### Biological read-out of the siRNA screen

The aim of our siRNA screen was to identify new components of the machinery responsible for the uptake and intracellular trafficking of H_C_T and p75^NTR^, with regulators of axonal retrograde transport being of particular interest for us. However, screening for changes in axonal transport in motor neurons using an automated imaging approach is technically challenging since it would require adapting our established live cell axonal retrograde transport assay (Lalli et al., 2003; Bohnert and Schiavo, 2005; Deinhardt et al., 2006; Restani et al., 2012) to a 96-well plate format.

A simplified protocol was therefore used to monitor the somatic accumulation of H_C_T and p75^NTR^ in the cell body over time, as a proxy for the retrograde transport of these probes from the axonal network. Motor neurons were thus incubated with fluorescent H_C_T and an antibody directed against the extracellular domain of p75^NTR^ (αp75^NTR^) for 2 h, before removing the remaining surface-bound probes by acid washing and quantifying probe accumulation in the cell bodies of GFP-positive motor neurons. Whilst the endocytosis of H_C_T into the somato-dendritic compartment is very rapid, the onset of axonal transport of this probe takes considerably longer (Bohnert and Schiavo, 2005). We determined that 2 h was the ideal end point for our assay because this allowed sufficient time for H_C_T internalized at distal entry sites to be transported and accumulate in the soma (Supplementary Figure 3A).

The co-incubation of selected samples with EHNA, a known inhibitor of the dynein-mediated retrograde transport of H_C_T (Lalli et al., 2003), served as positive controls for the decreased intracellular accumulation of both probes (Figures 1C–E), which enter largely overlapping trafficking pathways (Lalli and Schiavo, 2002; Deinhardt et al., 2006). However, in contrast to the overt effect of EHNA on H_C_T accumulation, which was visible throughout the time frame of the assay (Supplementary Figure 3A), the effects of dynein inhibition on p75^NTR^ accumulation only became apparent after 90 min (Supplementary Figure 3B).

Notably, the internalization kinetics of αp75^NTR^ when incubated alone overlapped with the αp75^NTR^ accumulation profile when cells were co-incubated with this antibody together with H_C_T and EHNA (Supplementary Figure 3B). This result was not unexpected since it agrees with previous findings showing that in the absence of H_C_T or neurotrophins, p75^NTR^ enters a local fast recycling pathway, and as such does not undergo long-range dynein mediated transport (Deinhardt et al., 2007). Therefore, treatment of motor neurons with EHNA in the absence of H_C_T was not expected to alter αp75^NTR^ accumulation (see Discussion).

### siRNA screening for modulators of axonal retrograde transport and endocytic sorting

Having established a robust experimental platform for the siRNA screen, HB9-GFP motor neurons were then transfected with a custom-selected siRNA library targeting molecular motors, their adaptors and associated proteins, which have been reported to regulate motor activity (Supplementary Table 1A). To monitor knockdown efficiency under the experimental conditions used for the screen, we determined the down-regulation of VAMP2 in the three independent replicates assayed from the 96-well plates used for the actual screen. As shown in Supplementary Figure 4, VAMP2 knockdown in *Vamp2* siRNA treated wells was consistently 80% or greater.

**Figure 2:**
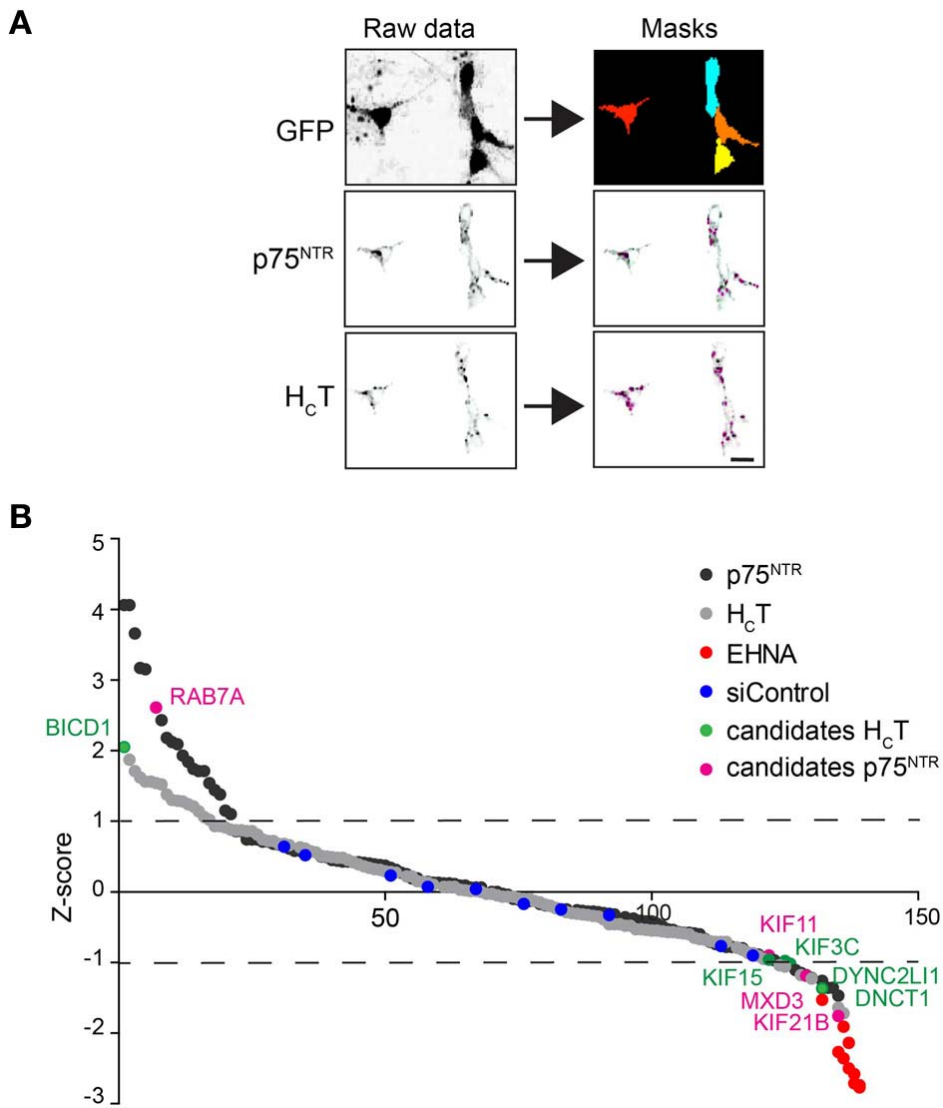
**Summary of the siRNA screen**. **(A)** Image analysis pipeline used for the siRNA screen. Representative images of GFP-positive motor neurons used to create image masks (top right panels), from which images of internalized H_C_T and p75^NTR^ were quantified. Scale bar, 10 μm. **(B)** Sigma plot of the Z-scores obtained for internalized H_C_T (gray circles) and p75^NTR^ (black circles) for the entire siRNA library (see Table S1). Candidates that produced statistically significant increased (Z-score: >1) or decreased (Z-score: <1) accumulation phenotypes are shown in green (H_C_T) or violet (p75^NTR^). EHNA-treated positive controls are shown in red.

The accumulation of H_C_T and αp75^NTR^ was quantified for all siRNAs by scoring the average number of H_C_T- and p75^NTR^-positive puncta per motor neuron using confocal microscopy followed by multi-parametric image analysis (Figure 2A) and represented as Z-scores (Figure 2B). Candidate hits were selected based on their standard deviations from the mean values for the whole siRNA library (see Materials and Methods and Supplementary Tables 1C,D) and are highlighted on the Z-score profile (Figure 2B).

From this primary analysis, 23 candidate genes for H_C_T and 28 for αp75^NTR^ were taken forward for validation using siRNA pools from a different supplier. The same assay was then performed for the re-screen except that hits were instead selected based on the standard deviation from the mean of the siRNA controls (Supplementary Tables 1E–G). RNAi of seven genes was confirmed to decrease probe accumulation, whereas only one gene for each probe was validated to induce the opposite effect (Supplementary Tables 1F,G). *Rab7* knockdown led to an increased intracellular accumulation of αp75^NTR^ (see Discussion), whilst the only silenced gene that was confirmed to boost the intracellular build-up of H_C_T was *Bicd1* (Figure 2B and Supplementary Tables 1F,G).

We decided to focus our efforts on addressing the role played by BICD1 in H_C_T trafficking in our model system since BICD1 and the related proteins BICD2 and BICDR1/2 have been described as microtubule-associated proteins and motor adaptors (Hoogenraad et al., 2001; Matanis et al., 2002; Schlager et al., 2010; Terenzio and Schiavo, 2010). Furthermore, BICD1 was proposed to play a role in axonal retrograde transport in neurons (Wanschers et al., 2007).

### The axonal retrograde transport of H_C_T is not affected in *Bicdl*^gt/gt^ motor neurons

Although RNA interference has emerged as a key strategy to analyse the function of mammalian genes and much work has centered on siRNA design to improve target specificity, individual siRNAs have nevertheless been shown to down-regulate unrelated genes by binding to the 3' untranslated regions of mRNAs resulting in off-target effects (Echeverri et al., 2006). To discount this possibility and confirm the specificity of the H_C_T accumulation phenotype induced by *Bicd1* knockdown, we independently assessed the effects of BICD1 depletion in motor neurons derived from an ES cell line engineered to carry a *Bicd1* gene trapped allele, *Bicd1*^gt/+^ (Terenzio et al., 2014). Motor neurons derived from ES cells homozygous for this mutation (*Bicd1*^gt/gt^) expressed 70% lower BICD1 protein compared to wild type cells. Importantly, *Bicd1*^gt/gt^ motor neurons were indistinguishable from *Bicd1*^gt/+^ and wild-type ES cell-derived motor neurons in terms of synaptic density or neurite differentiation (Terenzio et al., 2014). As such, *Bicd1*^gt/gt^ motor neurons are an ideal model to study the effects of BICD1 depletion on H_C_T trafficking.

**Figure 3:**
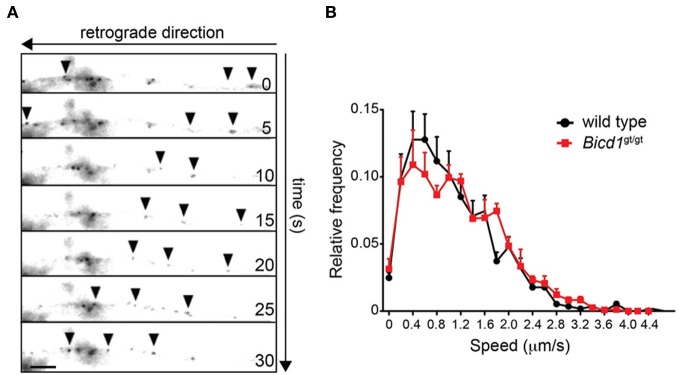
**Axonal retrograde transport of H_C_T is not perturbed in *Bicd1*^gt/gt^ motor neurons. (A)** Representative images taken from a time-lapse movie showing retrograde movement of BICD1-GFP (arrowheads) along the axon of a wild type motor neuron. Scale bar, 5 μm. **(B)** Speed distribution profiles of axonal AlexaFluor-conjugated H_C_T-positive carriers undergoing axonal retrograde transport in wild type (black squares) and *Bicd1*^gt/gt^ (red squares) motor neurons (mean ± s.e.m.).

Confirming the previous findings linking BICD1 to axonal transport (Wanschers et al., 2007), BICD1-GFP transfected into wild-type motor neurons underwent robust retrograde transport along the axonal network (Figure 3A). However, in spite of the reported interaction of BICD1 with cytoplasmic dynein, BICD1 does not appear to play a direct role in this pathway, since axonal retrograde transport of H_C_T was not perturbed in *Bicd1*^gt/gt^ motor neurons (Figure 3B).

### BICD1 depletion enhances the somatic accumulation of H_C_T

To confirm that the depletion of BICD1 in *Bicd1*^gt/gt^ motor neurons replicated the H_C_T accumulation phenotype identified during the siRNA screen, identical probe accumulation assays were performed using *Bicd1*^gt/+^ and *Bicd1*^gt/gt^ motor neurons. Thus, H_C_T and αp75^NTR^ were incubated together to trigger the internalization of p75^NTR^ via a clathrin-dependent route linked to axonal retrograde transport (Deinhardt et al., 2007). Probe internalization was quantified at 15, 30, and 60 min of incubation at 37°C, which demonstrated that the intracellular accumulation of both H_C_T and αp75^NTR^ was significantly increased in *Bicd1*^gt/gt^ motor neurons after 60 min (Supplementary Figure 5). This phenotype was also detected in *Bicd1*^gt/+^ cells, although it was statistically significant only for p75^NTR^ under these experimental conditions (Supplementary Figure 5B).

These data supported the results of our siRNA screen and further confirmed that depletion of BICD1 causes an increased somatic accumulation of H_C_T_._ However, a similar phenotype for the p75^NTR^ antibody was also observed in the *Bicd1*^gt/+^ model system, prompting us to further investigate the effects of BICD1 depletion on the trafficking of the neurotrophin receptors p75^NTR^ and TrkB, which are described elsewhere (Terenzio et al., 2014). Altogether, our data indicate that BICD1 functions as a regulator of the somatic sorting of signaling endosomes containing in H_C_T and neurotrophin receptors. However, this manuscript focuses primarily on investigating the regulatory role of BICD1 on H_C_T trafficking.

### BICD1 depletion caused the increased association of H_C_T with endosomal sorting compartments

**Figure 4:**
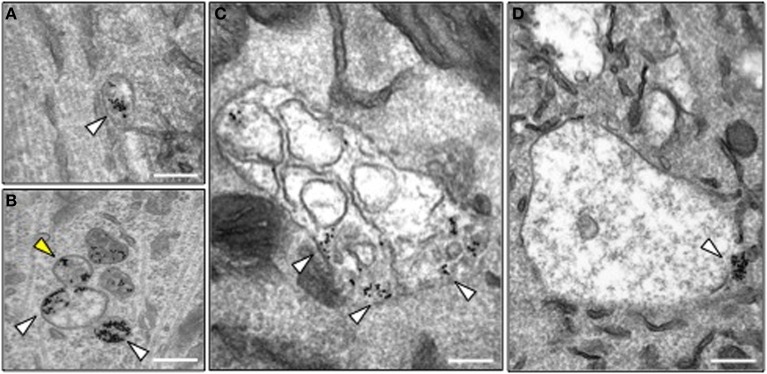
**Morphology of H_C_T-containing organelles in wild type and *Bicd1*^gt/gt^ motor neurons. (A)** Transmission electron microscopy image of a typical endosome containing gold-conjugated H_C_T (white arrowhead) in wild type motor neurons. H_C_T was internalized for 2 h at 37°C. Scale bar, 200 nm. **(B–D)** Transmission electron microscopy images of representative organelles containing gold-conjugated H_C_T (white arrowheads) in *Bicd1*^gt/gt^ motor neurons following internalization for 2 h at 37°C. H_C_T was often detected in budding structures (**B**, yellow arrowhead), or enlarged endosomal organelles containing membranes **(C)** or with an amorphous content **(D)**. In several instances, gold-conjugated H_C_T was found clustered at the limiting membrane of these structures. Scale bar, 200 nm.

In order to investigate the precise trafficking step affected by BICD1 depletion, we used transmission electron microscopy to trace the intracellular fate of internalized nanogold-conjugated H_C_T in wild type and *Bicd1*^gt/gt^ motor neurons. As previously reported, colloidal gold-conjugated H_C_T undergoes rapid internalization followed by axonal retrograde transport and accumulates in organelles resembling tubular endosomes and multivesicular bodies (MBV) (Parton et al., 1987; Lalli et al., 2003). Whilst this distribution pattern of H_C_T was confirmed in wild-type motor neurons (Figure 4A), BICD1 depletion induced a preferential accumulation in H_C_T and enlarged organelles with amorphous content, which were localized in the cell body of *Bicd1*^gt/gt^ motor neurons (Figures 4B–D). These organelles contained clustered gold-conjugated H_C_T, which was localized close to the limiting membrane and occasionally within buds or tubules emerging from these structures (Figure 4B).

**Figure 5:**
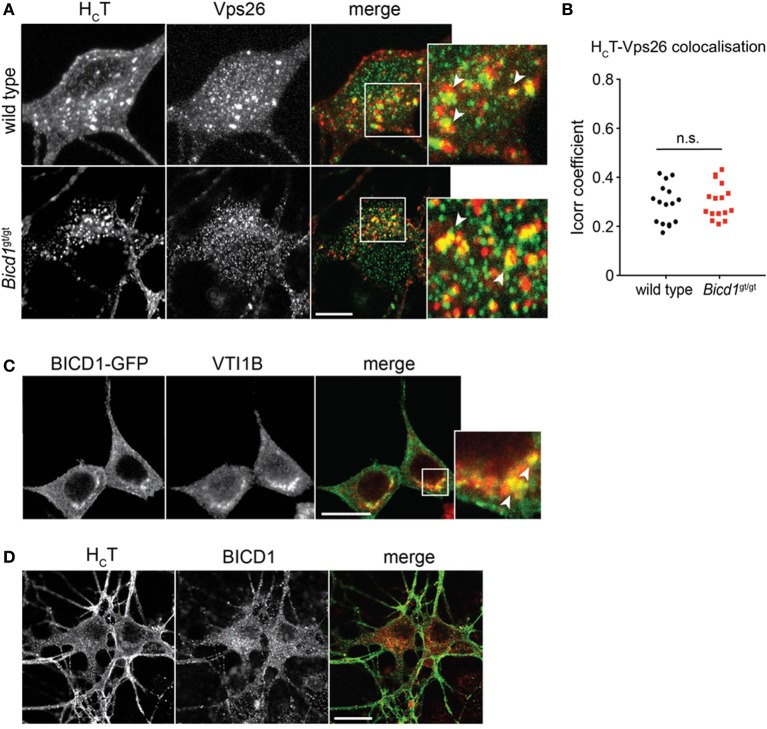
**H_C_T partially co-distributes with retromer components and late endosomal SNAREs. (A)** Wild type and *Bicd1*^gt/gt^ motor neurons were incubated with AlexaFluor555-conjugated H_C_T (red) for 1 h at 37°C, then acid-washed, fixed and immunostained for Vps26 (green). Insets from the merged images are magnified to show co-localization (yellow) between H_C_T and Vps26. Scale bar, 5 μm. **(B)** Quantification of H_C_T/Vps26 co-localization from the experiment shown in **(A)** (16 cells per condition, Mann-Whitney test, mean ± s.e.m., n.s. non-significant). **(C)** N2A cells over-expressing BICD1-GFP (green) were fixed and immunostained for VTI1B (red). The white box in the merged channel is magnified to highlight the extent of co-localization of BICD1-GFP and this late endosomal SNARE protein. Scale bar, 20 μm. **(D)** Wild type motor neurons were incubated with AlexaFluor488-conjugated H_C_T (green) for 1 h at 37°C, acid-washed, fixed and then immunostained for BICD1 (red). Co-localization between H_C_T and BICD1 was not detected under these conditions. Scale bar, 20 μm.

We believe that the organelles containing large clusters of gold-conjugated H_C_T represent enlarged endosomal sorting compartments. This view is supported by the observation that H_C_T co-localized with components of the retromer complex, such as Vps26 (Figures 5A,B) and sorting nexin 1 (SNX1) (Terenzio et al., 2014). Furthermore, BICD1 was found to partially co-distribute with selected endosomal markers, such as VTI1B (Figure 5C), a SNARE protein involved in late endosome to lysosome trafficking (Offenhauser et al., 2011). This result suggested that BICD1 might be important for this sorting route, which normally controls the down-regulation of ligand-activated receptor complexes. However, we never observed any co-localization between H_C_T and BICD1 (Figure 5D) or VTI1B (data not shown), suggesting either that the association of BICD1 with these markers was below the detection limits of our assays, or that BICD1 affected H_C_T behavior indirectly by altering endosomal sorting steps downstream of SNX1.

### BICD1 depletion increases H_C_T binding to the neuronal plasma membrane

**Figure 6:**
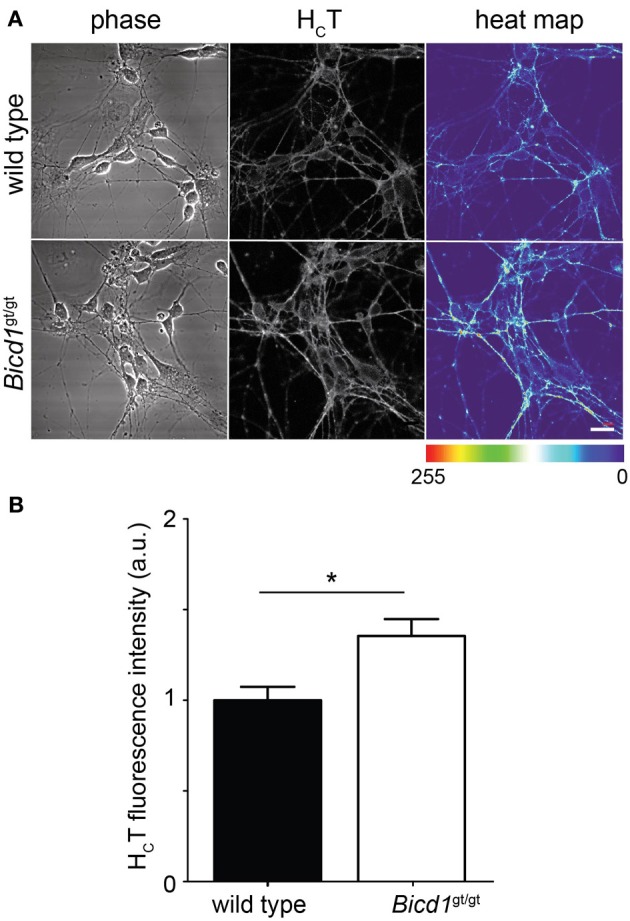
***Bicd1*^gt/gt^ motor neurons have increased cell surface binding of H_C_T**. **(A)** Wild type and *Bicd1*^gt/gt^ motor neurons were incubated on ice for 15 min with AlexaFluor555-conjugated H_C_T (grayscale) and fixed. Scale bar = 20 μm. A heat map representation of H_C_T staining has been added to better visualize the differences in binding between the two genotypes (right panels). **(B)** Quantification of the cell surface pool of H_C_T from three independent experiments (*t*-test, mean ± s.e.m., ^*^p < 0.05).

The retromer complex is an essential component of the endosomal protein sorting machinery, which regulates the retrieval of cargoes in transit through the endosomal pathway to the Golgi or their re-targeting back to the plasma membrane. Misregulation of retromer-mediated sorting impairs these processes and causes severe neurological diseases, such as hereditary spastic paraplegia (Seaman, 2012). Whereas targeting to the Golgi was unaffected by BICD1 depletion (Terenzio et al., 2014 and data not shown), the binding of H_C_T to the plasma membrane was significantly enhanced in *Bicd1*^gt/gt^ motor neurons compared to wild-type cells (Figure 6). Similarly, TrkB and p75 also showed increased accumulation on the plasma membrane of mutant neurons (Terenzio et al., 2014).

## Discussion

In this work, we have developed a novel siRNA screening approach designed to identify new players involved in the internalization and trafficking of tetanus toxin and neurotrophin receptor complexes in motor neurons. We designed an imaging-based assay using a 96-well plate format, which allowed us to screen a custom-selected siRNA library for genes affecting the trafficking of neurotropic probes. Important considerations for protocol design included the selection of appropriate fluorescent probes and a relevant cell model to monitor their intracellular trafficking.

To this end, we chose H_C_T because of our long-standing interest in studying the molecular mechanisms responsible for the binding, internalization and intracellular trafficking of tetanus neurotoxin, which continues to pose a serious threat to human health in the developing world. The second probe used in this study was an antibody against the extracellular domain of p75^NTR^, which has previously been shown to act as a reliable tool to monitor the internalization and long-range transport of p75^NTR^ in a ligand-dependent manner (Deinhardt et al., 2007). Upon addition of neurotrophins, this antibody enters signaling endosomes containing H_C_T and Trk receptors, which are retrogradely transported along the axon (Deinhardt et al., 2006).

Motor neurons are the most physiologically relevant model system with which to perform such a screen. We used HBG3 ES cells for differentiating large numbers of GFP-expressing motor neurons, which could be easily imaged for the quantification of fluorescent probe accumulation.

One aspect of the screen that underwent extensive optimization was the timing of internalization of the two probes. We performed a kinetic analysis of H_C_T and p75^NTR^ internalization (Supplementary Figure 3) and established that the two probes behaved differently in their response to the cytoplasmic dynein inhibitor EHNA, which was used in our screen as a positive control for inhibition of axonal transport. EHNA inhibited the accumulation of H_C_T, even from the earliest time points, whereas this inhibitor affected p75^NTR^ accumulation only after 90 min. A possible explanation for this phenomenon is that in the absence of neurotrophins, p75^NTR^ enters a local recycling pathway and does not undergo long-range axonal retrograde transport. Hence, treatment with EHNA would not be expected to change p75^NTR^ accumulation in the absence of any cognate ligands. However, H_C_T is known to promote p75^NTR^ axonal transport (Deinhardt et al., 2007), thereby increasing the delivery of this receptor to the soma. This may be the most likely explanation for the increased p75^NTR^ accumulation in the presence of H_C_T and that EHNA-induced effects for p75 were only noted from 90 min onwards.

Our screen identified several genes that affected the internalization and/or accumulation of H_C_T and αp75^NTR^. For example, the knockdown of Rab7 increased the intracellular accumulation of p75^NTR^ (Figure 2B and Supplementary Table 1G), possibly due to the defective progression of this receptor along the endosomal pathway, a process known to be regulated by Rab7 (Stenmark, 2009; Mizuno-Yamasaki et al., 2012). This result is in agreement with the accumulation of NGF-activated TrkA in enlarged endosomes in PC12 cells overexpressing a dominant-negative Rab7 mutant (Saxena et al., 2005). Although our screen was performed in the absence of exogenous neurotrophins, H_C_T has been found to activate neurotrophin signaling in a Trk-dependent manner (Calvo et al., 2012), and therefore may direct p75^NTR^ toward a long-range axonal trafficking pathway, which is normally only followed by ligand-activated receptors (Deinhardt et al., 2007).

Since the uptake and axonal transport of H_C_T and p75^NTR^ share a common route (Bercsenyi et al., 2013; Schmieg et al., 2013), it was perhaps surprising that our screen failed to identify any gene equally affecting the intracellular dynamics of both probes. For instance, the cytoplasmic dynein/dynactin complex is known to play a key role in the axonal retrograde transport of both H_C_T and p75^NTR^ (Bercsenyi et al., 2013). Of the many cytoplasmic dynein and dynactin genes that we screened, only the knockdown of dynein light chain Tctex-3 (*Dynlt3*) and p150^*Glued*^ (*Dcnt1*) reduced the accumulation of H_C_T without affecting intracellular increase of αp75^NTR^ (Figure 2B and Supplementary Tables 1F,G). One possible explanation for this unexpected result is that the internalization and transport of H_C_T and p75^NTR^ are differentially sensitive to the degree of silencing of genes that regulate their trafficking, such as dynein/dynactin subunits or specific Rab GTPases. Decreased accumulation phenotypes were more common for anterograde kinesin heavy chains, such as KIF11, KIF15, and KIF21B (Figure 2B and Supplementary Tables 1F,G). Experiments aimed at addressing the function of these kinesins in neurotrophin receptor trafficking will be the focus of future studies.

Since tetanus toxin traffics to the central nervous system by dynein-mediated retrograde transport and BICD1 is a known cytoplasmic dynein adaptor, we concentrated our efforts on investigating how BICD1 depletion affected the accumulation of H_C_T in motor neurons. Our results suggest that BICD1 regulates H_C_T trafficking, which may provide insights into how this dynein adaptor might influence tetanus toxin behavior *in vivo*. Furthermore, BICD1 is known to play diverse roles in the development and maintenance of the nervous system (Matanis et al., 2002; Schlager et al., 2010) yet its mechanism of action in this context has not been established. Because BICD1 was proposed to play a role in axonal retrograde transport (Wanschers et al., 2007) and is transported along axons (Figure 3A), we postulated that BICD1 depletion might interfere with long-range axonal trafficking of signaling endosomes containing H_C_T and p75^NTR^. However, the axonal retrograde transport of H_C_T was not perturbed in *Bicd1*^gt/gt^ motor neurons (Figure 3B). Similarly, axonal trafficking of p75^NTR^ was also unaffected in *Bicd1*^gt/gt^ motor neurons upon BDNF stimulation (Terenzio et al., 2014), suggesting that either BICD1 is not essential for the long-range axonal trafficking of H_C_T and p75^NTR^, or that the residual BICD1 protein level in *Bicd1*^gt/gt^ motor neurons is sufficient to support the recruitment of cytoplasmic dynein to H_C_T-containing signaling endosomes and/or the regulation of dynein activity associated with these endosomes.

Recently, the mechanisms responsible for cargo sorting within signaling endosomes and the fate of ligand-receptor complexes upon arrival in the cell body have gained mounting interest. Based on our analysis of H_C_T trafficking, we hypothesized that BICD1 was likely to have a role in the somatic sorting of this probe. In support of this view, electron microscopy analyses performed in motor neurons depleted of BICD1 demonstrated that gold-conjugated H_C_T exhibited a higher association with enlarged membrane compartments, where it clustered close to the limiting membrane, sometimes within bud-like structures connected to these organelles (Figure 4). This finding suggested that BICD1 is involved in the regulation of the endosomal trafficking of H_C_T and neurotrophin receptor complexes and that its depletion alters cargo flow within the endosomal pathway.

The retromer complex is a crucial component of the endosomal sorting machinery, and plays major roles in endosome to Golgi retrieval and endosome to plasma membrane recycling processes, through interactions with members of the sorting nexin (SNX) family (Seaman, 2012). Recently, more than 100 plasma membrane proteins were found to bind to SNX27, including the neurotrophin receptor scaffolding protein Kidins220/ARMS (Steinberg et al., 2013). Importantly, SNX27 is required to maintain plasma membrane levels of these proteins by promoting their recycling and preventing their degradation (Steinberg et al., 2013). We found that H_C_T colocalizes with retromer components, such as Vps26 (Figure 5A), and SNX1 (Terenzio et al., 2014). Crucially, the co-distribution of H_C_T with SNX1-positive compartments was higher in *Bicd1*^gt/gt^ motor neurons compared to wild type cells, suggesting that SNX1-dependent sorting of H_C_T and associated cargoes was affected by BICD1 depletion (Terenzio et al., 2014).

Supporting our proposed role for BICD1 in endosomal sorting, depletion of BICD1 increased plasma membrane levels of H_C_T (Figure 6) as well as the neurotrophin-dependent recycling of p75^NTR^ and TrkB to the plasma membrane (Terenzio et al., 2014). At the present time, we do not have a molecular explanation for the increased cell surface binding of H_C_T by *Bicd1*^gt/gt^ motor neurons, but it is likely to relate to increased toxin receptor levels at the plasma membrane. Even though the protein receptor of H_C_T is not known (Bercsenyi et al., 2013), this probe co-localizes with TrkB, undergoes axonal retrograde transport with this receptor (Deinhardt et al., 2006) and activates signaling cascades downstream of Trks in cortical and hippocampal neurons (Gil et al., 2000, 2003; Calvo et al., 2012).

**Figure 7:**
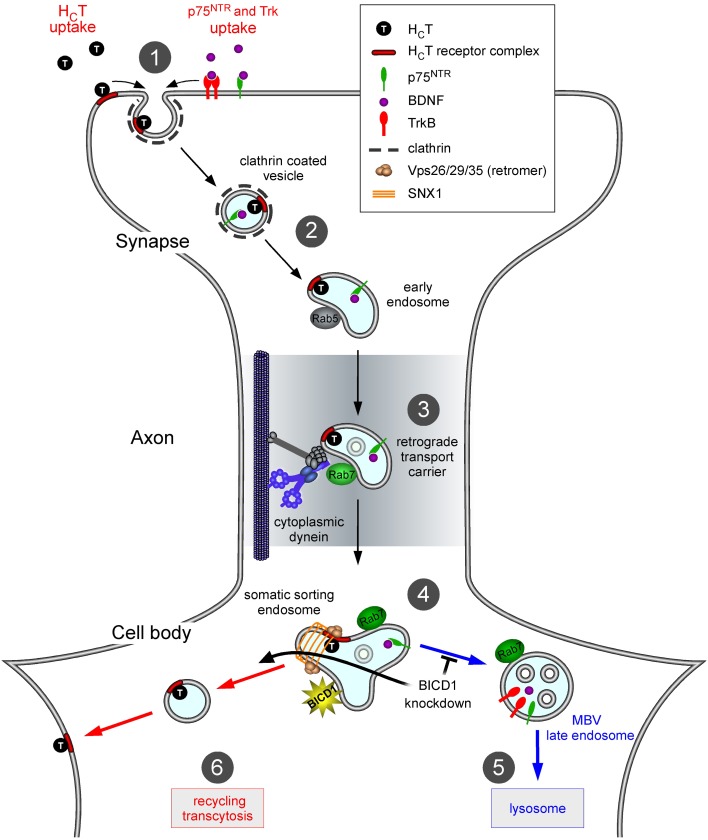
**Proposed role of BICD1 in H_C_T trafficking**. In wild type motor neurons, H_C_T binds to plasma membrane receptors, which include polysialogangliosides and is internalized at synaptic sites located in the periphery **(1)**; note that for clarity, internalization of H_C_T along the axon and in the soma is not shown. H_C_T and neurotrophin-receptor complexes are sorted to signaling endosomes **(2)**, which are retrogradely transported by cytoplasmic dynein **(3)**, toward the cell soma. Here, they associate with somatic sorting endosomes **(4)** decorated by sorting nexin 1 (SNX1) and other retromer components. Neurotrophin receptors are then trafficked toward MVB/lysosomes **(5)** for degradation, whilst H_C_T is recycled to the plasma membrane **(6)**. Impairment of the lysosomal targeting of TrkB and other proteins, including H_C_T receptors, in neurons lacking BICD1 is envisaged to mainly redirect them to the recycling route back to the plasma membrane. The main consequence of these miss-sorting steps is an increase in H_C_T binding sites and neurotrophin receptors (Terenzio et al., 2014) on the cell surface at steady state.

Based on these results, we would like to propose a model by which BICD1 controls the sorting of cargoes within axonal signaling endosomes and subsequently directs them to appropriate degradation or recycling routes (Figure 7). This role, which is required for optimal neurotrophin signaling (Terenzio et al., 2014), is also likely to control the trafficking of pathogens and virulence factors, such as tetanus neurotoxin and its binding fragment H_C_T, which exploit axonal signaling endosomes for their entry and dissemination in the nervous system (Salinas et al., 2010; Schmieg et al., 2013). In this light, modulating BICD1 activity could have potential therapeutic effects on the controlling the infectivity and/or spreading of viruses and toxins, and may be exploited to improve the efficiency of gene therapy vectors targeting the nervous system.

## Author contributions

All authors provided substantial contributions to this work. In particular, Marco Terenzio and Giampietro Schiavo contributed to the conception, experimental design, data interpretation and drafting of the manuscript. Marco Terenzio contributed to the setup of the ES differentiation protocols and to data analysis. All authors contributed to manuscript revision and approval of the final version. All authors are accountable in ensuring that questions related to the accuracy or integrity of any part of the work are appropriately investigated and resolved.

### Conflict of interest statement

The authors declare that the research was conducted in the absence of any commercial or financial relationships that could be construed as a potential conflict of interest.

## Supplementary material

Supplementary Figure 1 **Optimization of the transfection protocol. (A)** VAMP2 knockdown in primary motor neurons using different transfection reagents in a 96 well plate format. The spot count for VAMP2-positive vesicles for the reagents that proved non-toxic (12 out of the original 23) are shown. 2 different VAMP2 siRNA concentrations, 50 and 100 nM, were used for each reagent. **(B)** The most effective eight reagents from **(A)** were re-screened using the same protocol, but using three different concentrations of each reagent. The quantity of each reagent used is indicated. Samples treated with the indicated reagent and the siRNA control pool or the VAMP2 siRNA pool are indicated by S or V, respectively (*n* = 3). **(C)** The most effective four reagents from panel B were tested for their ability to induce knockdown of VAMP2. Western blotting of cell lysates demonstrated that all four reagents proved to be effective in downregulating VAMP2.

Supplementary Figure 2 **Derivation of motor neurons from mouse embryonic stem cells. (A)** Flow diagram describing the protocol used for the differentiation of motor neurons from mouse HB9-GFP ES cells. After growth in suspension in differentiating medium for 3 days, the ES cells form embryoid bodies (EBs) comprising neuronal progenitor cells (NPC). These NPCs are then induced to commit to a motor neuron fate (indicated by GFP expression) by the addition of retinoic acid (RA) and Smoothened Agonist (SAG), a small molecule activator of sonic hedgehog signaling. At the end of the fourth day in the presence of these differentiating agents, the EBs are dissociated and motor neurons plated. Scale bar, 10 μm. **(B)** HB9-GFP ES cell derived motor neurons (GFP, green) 4 days after plating and immunostained for GFP (red) and the pan neuronal marker, βIII tubulin (blue). Note the characteristically long motor neuron axon (arrowhead), arising for the soma (asterisk). Scale bar, 50 μm.

Supplementary Figure 3 **Kinetics of H**_C_**T** **and p75**^NTR^ **antibody internalization. (A)** H_C_T accumulation kinetics in motor neurons derived from HB9-GFP ES cells in the presence of αp75^NTR^ and the presence (red) or absence (black) of EHNA (*n* = 3). Note that H_C_T accumulation was significantly decreased in the presence of EHNA (*p* < 0.0001, Two-Ways ANOVA). **(B)** Accumulation kinetics of the p75^NTR^ antibody (αp75^NTR^) when co-incubated with H_C_T in motor neurons derived from HB9-GFP ES cells in the absence (black) or presence (red) of EHNA (*n* = 3). Note that the receptor accumulation profiles generated from EHNA-treated samples diverge from the control plot only after 75 min (*p* < 0.0001, Two-Ways ANOVA). Importantly, in the absence of H_C_T, the internalization kinetics of the p75^NTR^ antibody (blue) overlapped with the internalization profile observed for this probe when it was co-incubated with H_C_T and EHNA.

Supplementary Figure 4 **Assessment of knockdown efficiency of the siRNA screen**. Five wells from each plate of the siRNA screen were treated with non-targeting control siRNA and five wells with VAMP2 siRNA. Protein lysates from each set of samples for each plate were pooled and analyzed by western blot to assess VAMP2 knockdown efficiency with βIII tubulin serving as a loading control.

Supplementary Figure 5 **Internalization kinetics of H**_C_**T** **and p75**^NTR^ **antibody in wild type, *Bicd1***^gt/+^ **and *Bicd1***^gt/gt^ **motor neurons. (A)** H_C_T internalization kinetics were assessed in wild type, *Bicd1*^gt/+^ and *Bicd1*^gt/gt^ motor neurons. At the 60 min time point, a statistically significant increase in H_C_T accumulation was confirmed for *Bicd1*^gt/gt^ motor neurons compared to wild type cells. (ANOVA test, followed by Bonferroni post-test, *n* = 3. Results are shown ± s.e.m.). **(B)** αp75^NTR^ antibody internalization kinetics were assessed in wild type, *Bicd1*^gt/+^ and *Bicd1*^gt/gt^ motor neurons. A statistically significant difference between the three genotypes was observed at the 60 min time point. Statistical significance was calculated using a One-Way ANOVA test, followed by Bonferroni post-test, *n* = 3. Results are shown ± s.e.m.
