# Supplementary figures and images for: siRNA screen of ES cell-derived motor neurons identifies novel regulators of tetanus toxin and neurotrophin receptor trafficking
Source: Front Cell Neurosci. 2014 May 20;8:140. doi: 10.3389/fncel.2014.00140 (PMC4033017; doi:10.3389/fncel.2014.00140)

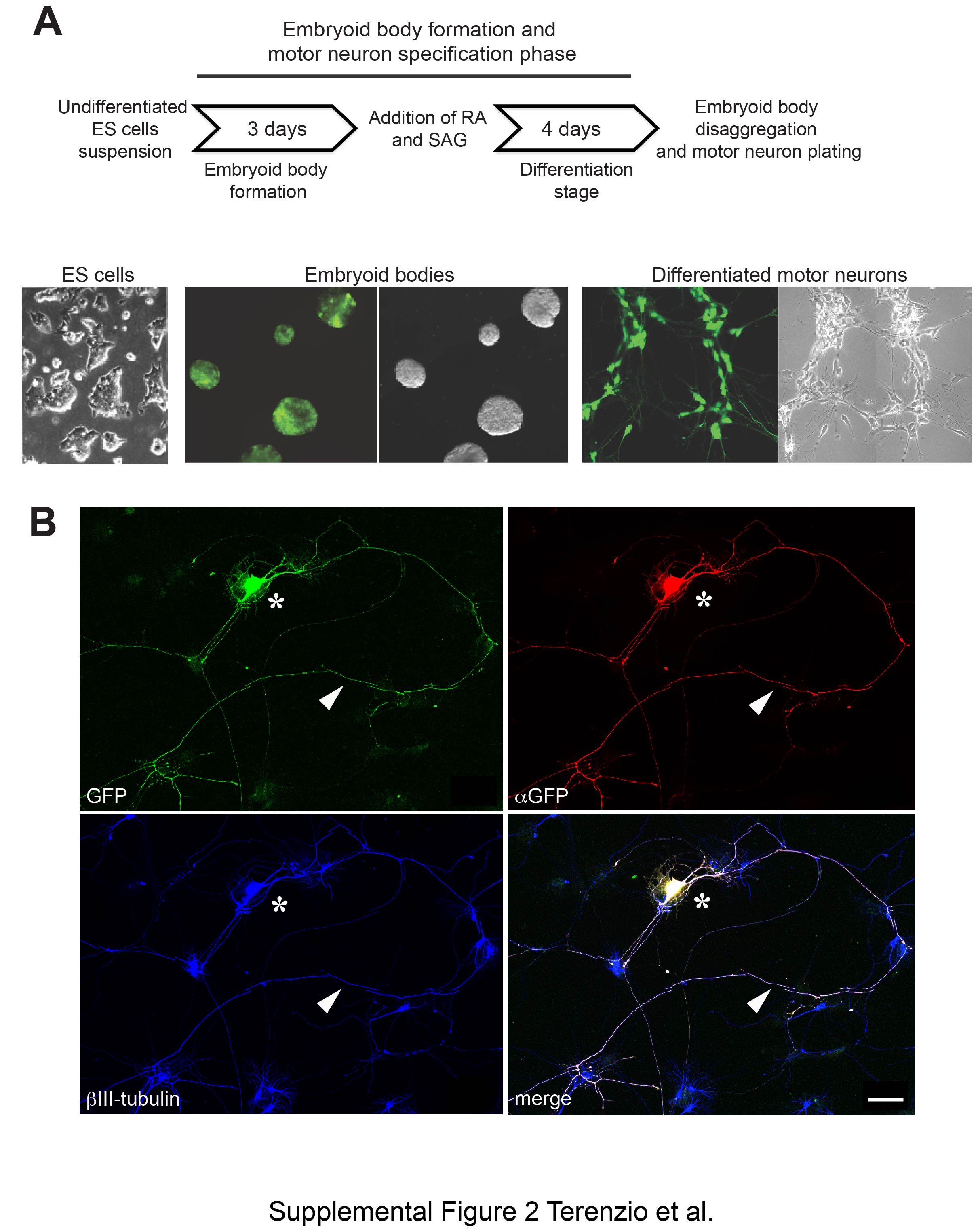

Supplement: Supplementary Figure 1 — Optimization of the transfection protocol. (A) VAMP2 knockdown in primary motor neurons using different transfection reagents in a 96 well plate format. The spot count for VAMP2-positive vesicles for the reagents that proved non-toxic (12 out of the original 23) are shown. 2 different VAMP2 siRNA concentrations, 50 and 100 nM, were used for each reagent. (B) The most effective eight reagents from (A) were re-screened using the same protocol, but using three different concentrations of each reagent. The quantity of each reagent used is indicated. Samples treated with the indicated reagent and the siRNA control pool or the VAMP2 siRNA pool are indicated by S or V, respectively (n = 3). (C) The most effective four reagents from panel B were tested for their ability to induce knockdown of VAMP2. Western blotting of cell lysates demonstrated that all four reagents proved to be effective in downregulating VAMP2. [file Presentation1.ZIP › 89038_Schiavo_Supp fig 2.JPEG]

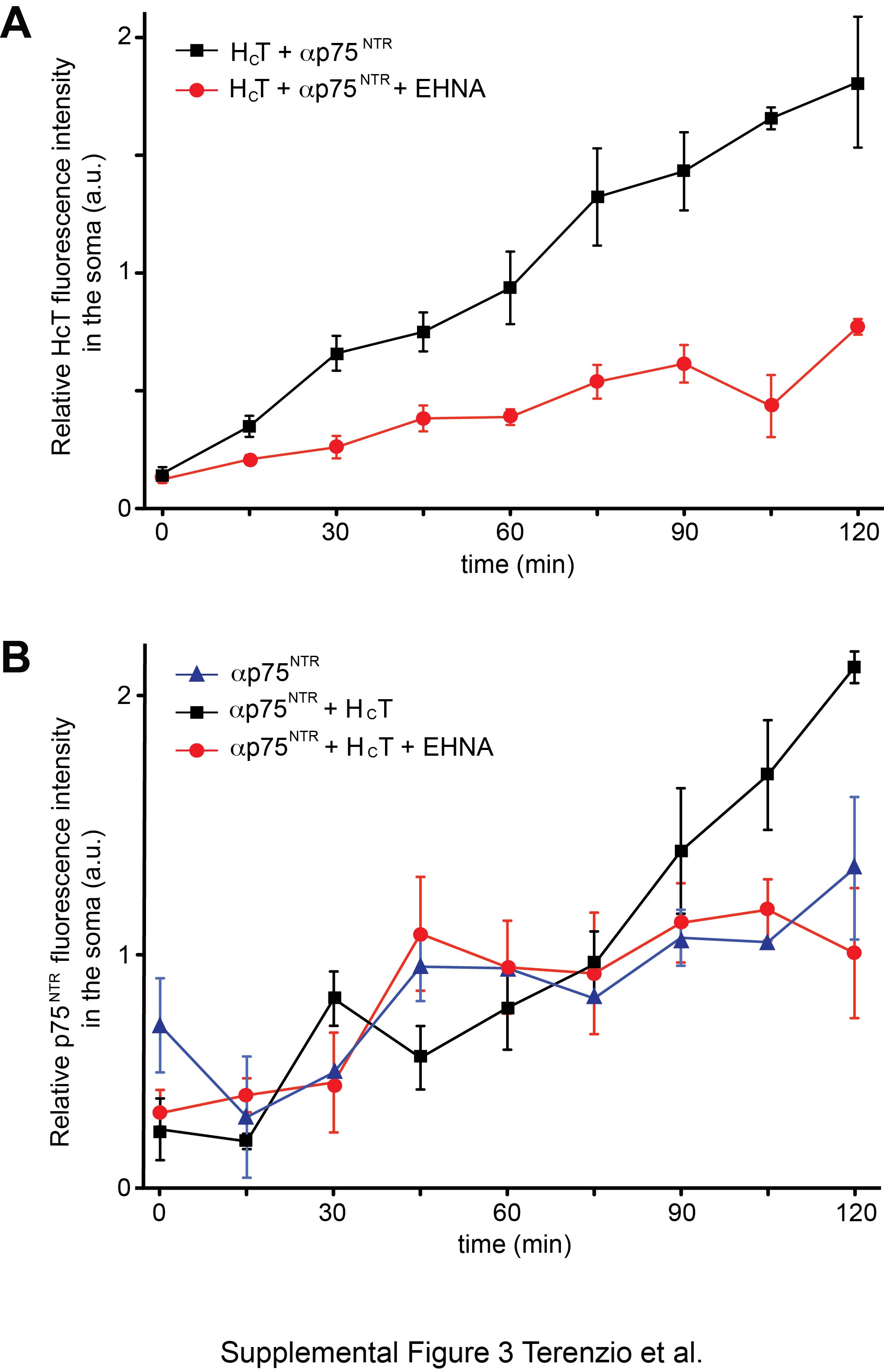

Supplement: Supplementary Figure 1 — Optimization of the transfection protocol. (A) VAMP2 knockdown in primary motor neurons using different transfection reagents in a 96 well plate format. The spot count for VAMP2-positive vesicles for the reagents that proved non-toxic (12 out of the original 23) are shown. 2 different VAMP2 siRNA concentrations, 50 and 100 nM, were used for each reagent. (B) The most effective eight reagents from (A) were re-screened using the same protocol, but using three different concentrations of each reagent. The quantity of each reagent used is indicated. Samples treated with the indicated reagent and the siRNA control pool or the VAMP2 siRNA pool are indicated by S or V, respectively (n = 3). (C) The most effective four reagents from panel B were tested for their ability to induce knockdown of VAMP2. Western blotting of cell lysates demonstrated that all four reagents proved to be effective in downregulating VAMP2. [file Presentation1.ZIP › 89038_Schiavo_Supp Fig 3.JPEG]

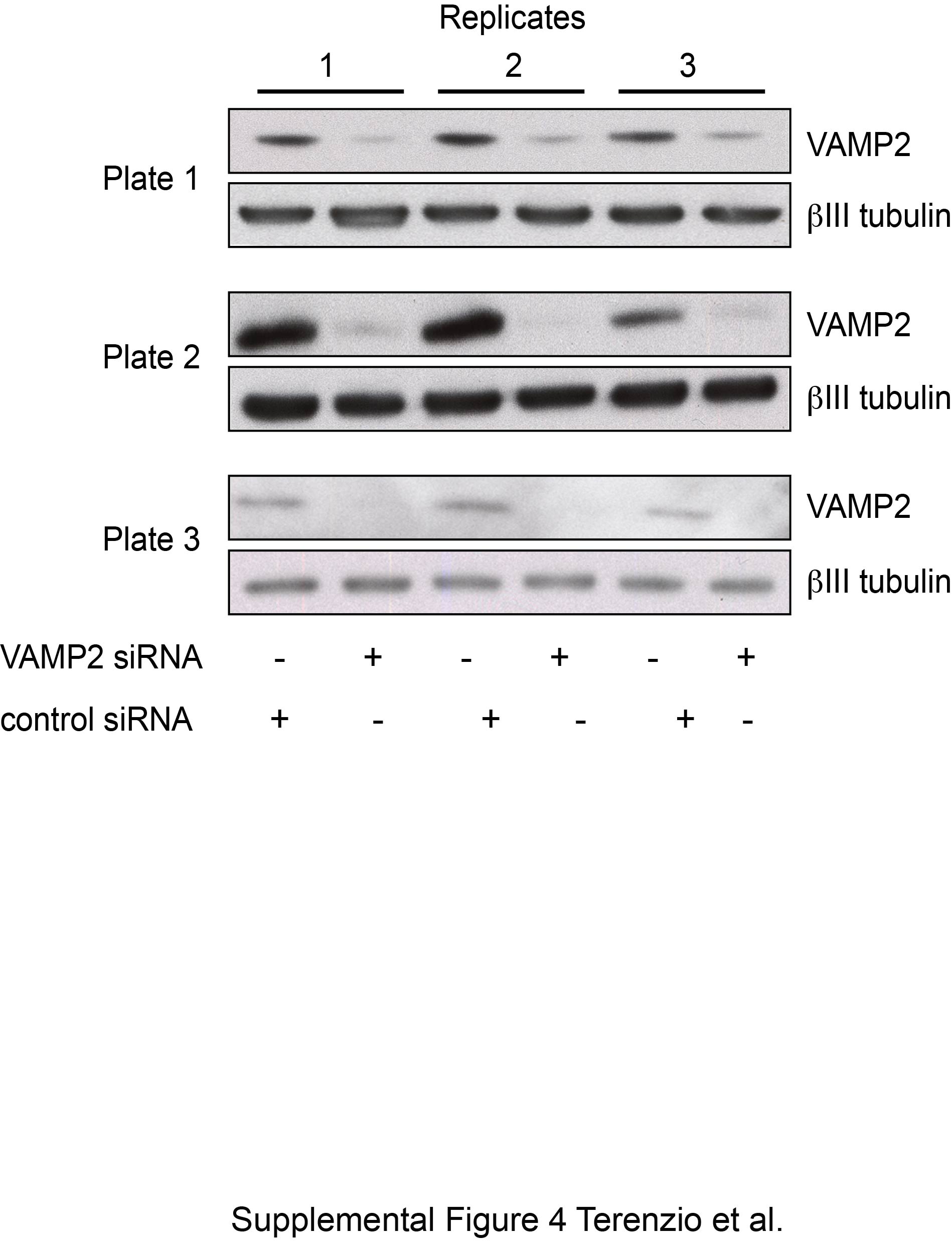

Supplement: Supplementary Figure 1 — Optimization of the transfection protocol. (A) VAMP2 knockdown in primary motor neurons using different transfection reagents in a 96 well plate format. The spot count for VAMP2-positive vesicles for the reagents that proved non-toxic (12 out of the original 23) are shown. 2 different VAMP2 siRNA concentrations, 50 and 100 nM, were used for each reagent. (B) The most effective eight reagents from (A) were re-screened using the same protocol, but using three different concentrations of each reagent. The quantity of each reagent used is indicated. Samples treated with the indicated reagent and the siRNA control pool or the VAMP2 siRNA pool are indicated by S or V, respectively (n = 3). (C) The most effective four reagents from panel B were tested for their ability to induce knockdown of VAMP2. Western blotting of cell lysates demonstrated that all four reagents proved to be effective in downregulating VAMP2. [file Presentation1.ZIP › 89038_Schiavo_Supp Fig 4.JPEG]

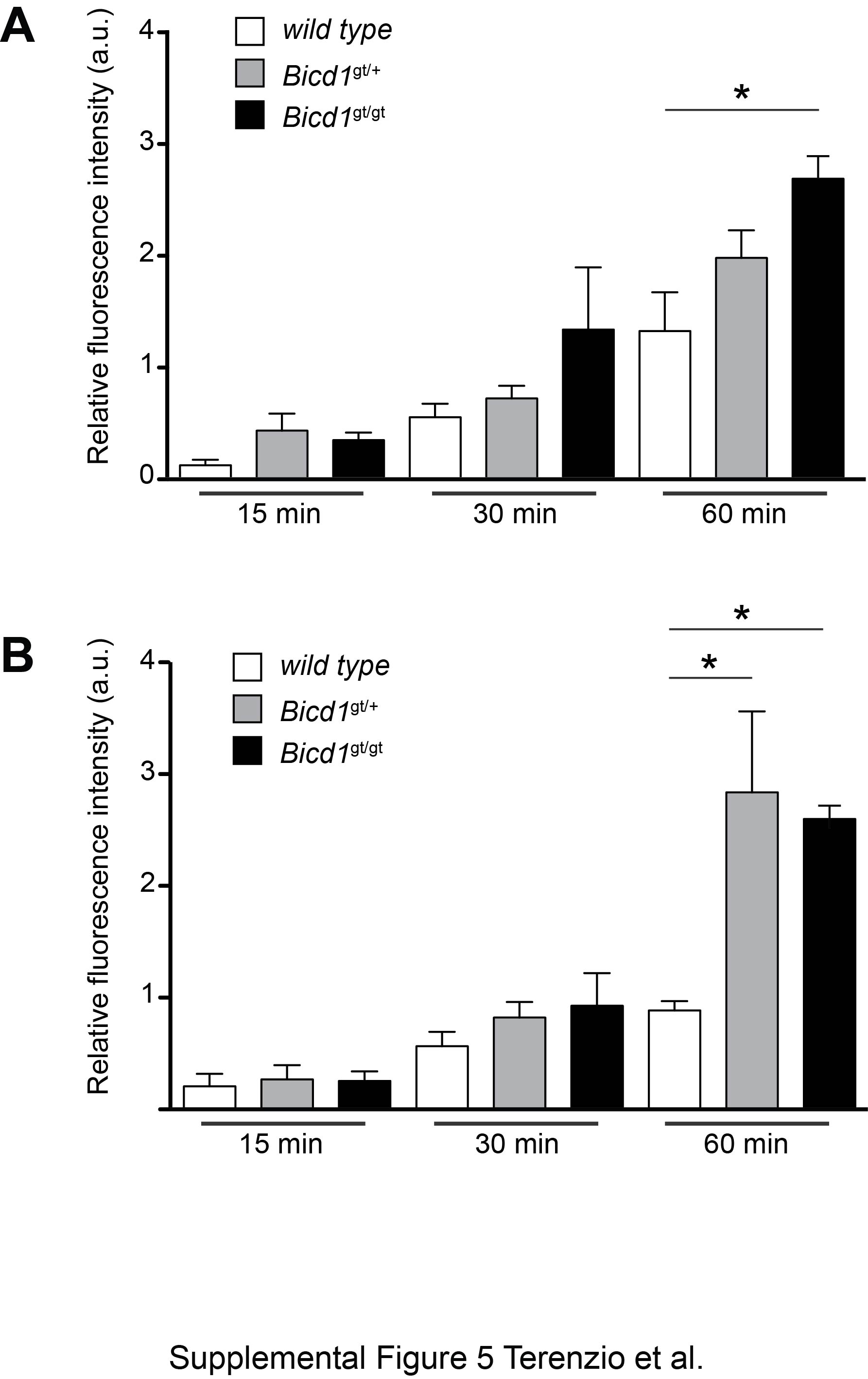

Supplement: Supplementary Figure 1 — Optimization of the transfection protocol. (A) VAMP2 knockdown in primary motor neurons using different transfection reagents in a 96 well plate format. The spot count for VAMP2-positive vesicles for the reagents that proved non-toxic (12 out of the original 23) are shown. 2 different VAMP2 siRNA concentrations, 50 and 100 nM, were used for each reagent. (B) The most effective eight reagents from (A) were re-screened using the same protocol, but using three different concentrations of each reagent. The quantity of each reagent used is indicated. Samples treated with the indicated reagent and the siRNA control pool or the VAMP2 siRNA pool are indicated by S or V, respectively (n = 3). (C) The most effective four reagents from panel B were tested for their ability to induce knockdown of VAMP2. Western blotting of cell lysates demonstrated that all four reagents proved to be effective in downregulating VAMP2. [file Presentation1.ZIP › 89038_Schiavo_Supp Fig 5.JPEG]

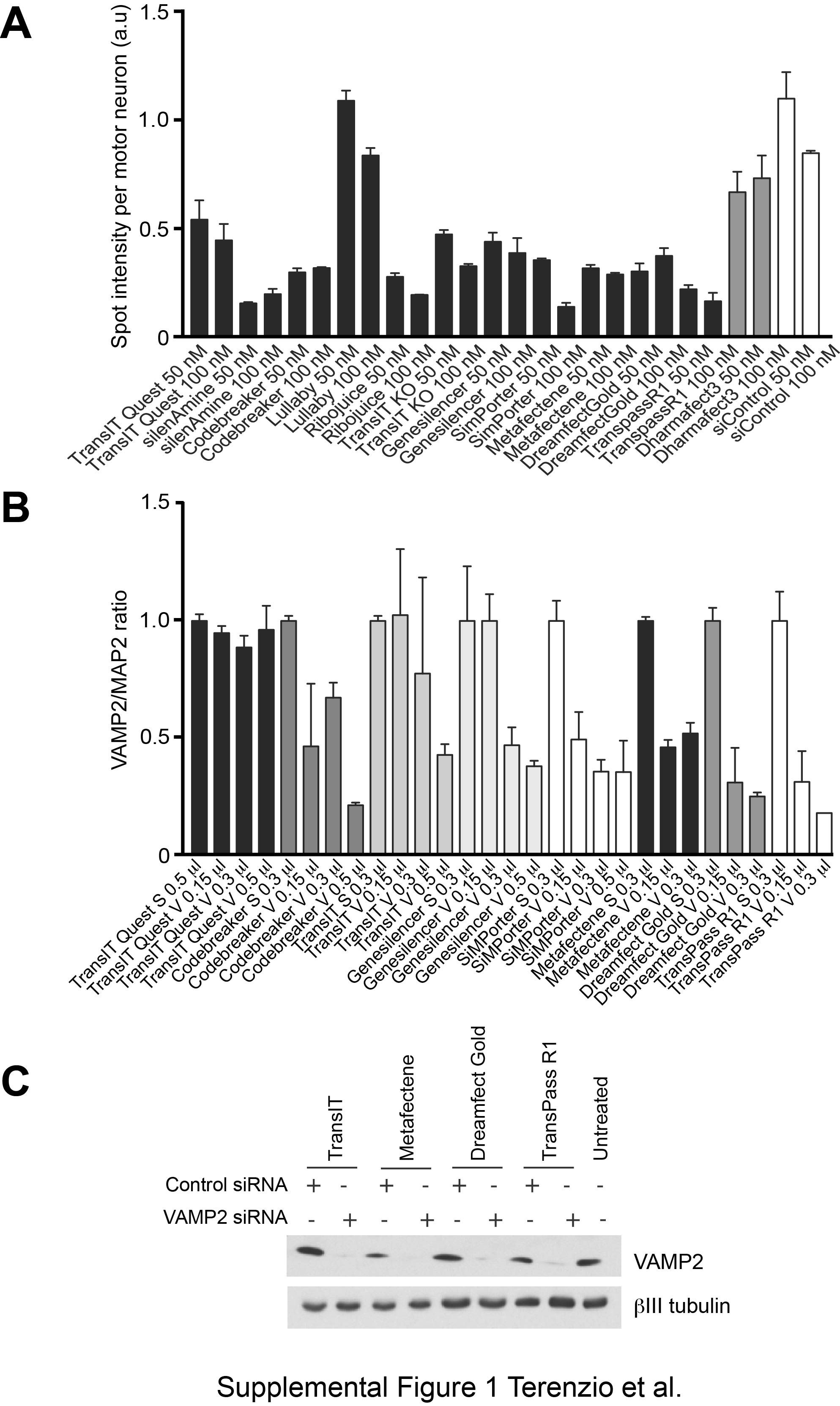

Supplement: Supplementary Figure 1 — Optimization of the transfection protocol. (A) VAMP2 knockdown in primary motor neurons using different transfection reagents in a 96 well plate format. The spot count for VAMP2-positive vesicles for the reagents that proved non-toxic (12 out of the original 23) are shown. 2 different VAMP2 siRNA concentrations, 50 and 100 nM, were used for each reagent. (B) The most effective eight reagents from (A) were re-screened using the same protocol, but using three different concentrations of each reagent. The quantity of each reagent used is indicated. Samples treated with the indicated reagent and the siRNA control pool or the VAMP2 siRNA pool are indicated by S or V, respectively (n = 3). (C) The most effective four reagents from panel B were tested for their ability to induce knockdown of VAMP2. Western blotting of cell lysates demonstrated that all four reagents proved to be effective in downregulating VAMP2. [file Presentation1.ZIP › 89038_Schiavo_Sup fig1.JPEG]
