# Supplementary material for: siRNA screen of ES cell-derived motor neurons identifies novel regulators of tetanus toxin and neurotrophin receptor trafficking
Source: Front Cell Neurosci. 2014 May 20;8:140. doi: 10.3389/fncel.2014.00140 (PMC4033017; doi:10.3389/fncel.2014.00140)
Supplement: Supplementary file 2 [file DataSheet1.DOCX]

**Supplementary Table 1.**

**Summary of the primary and validation siRNA screens**

(A) 96-well plate layout listing the positions on the master plate, gene IDs, symbols and names of targeted genes contained in the Qiagen siRNA library used in the primary screen.

(B) siRNA targeted cDNA sequences for all genes and non-targeting controls contained in the siRNA library used in the primary screen.

(C, D) Z-scores obtained in the primary screen. The Z-scores from the three replicates of each well are indicated for H_C_T (C) and αp75^NTR^ (D). The “well annotation” column indicates whether the well was treated with a targeting siRNA (sample), a non-targeting siRNA control (siControls) or a positive control for the assay (EHNA-treated samples). siControls are highlighted in grey and EHNA treated samples are in red.

(E) Plate layout of the validation re-screen. Position on the master plate, symbol, gene ID, and the accession number of the gene targeted are listed for each of the siRNA pool of the re-screen library.

(F, G) Validation re-screen scores. The score for each candidate for H_C_T (F) and αp75^NTR^ (G) is indicated together with the value obtained by subtracting the median of the siRNA controls from the individual score for each gene. The scores of the siControls and the EHNA-treated samples are highlighted in grey. The scores are obtained by calculating the mean of the five replicates for each candidate gene.

Supplementary Table 1

**A) Plate layout**

| **PLATE 1** | | | | |
| --- | --- | --- | --- | --- |
| **row** | **col** | **geneId** | **symbol** | **description** |
| B | 2 | 16560 | ***Kif1a*** | kinesin family member 1A |
| B | 3 | 12121 | ***Bicd1*** | bicaudal D homolog 1 |
| B | 4 | 229841 | ***Cenpe*** | centromere protein E |
| B | 5 | 13191 | ***Dctn1*** | dynactin 1 |
| B | 6 | 13424 | ***Dync1h1*** | dynein cytoplasmic 1 heavy chain 1 |
| B | 7 | 13426 | ***Dync1i1*** | dynein cytoplasmic 1 intermediate chain 1 |
| B | 8 | 13427 | ***Dync1i2*** | dynein cytoplasmic 1 intermediate chain 2 |
| B | 9 | 234663 | ***Dync1li2*** | dynein, cytoplasmic 1 light intermediate chain 2 |
| B | 10 | 16563 | ***Kif2a*** | kinesin family member 2A |
| B | 11 | 16570 | ***Kif3c*** | kinesin family member 3C |
| C | 2 | 16574 | ***Kif5c*** | kinesin family member 5C |
| C | 3 | 16573 | ***Kif5b*** | kinesin family member 5B |
| C | 4 | 17300 | ***Foxc1*** | forkhead box C1 |
| C | 5 | 16582 | ***Kifc3*** | kinesin family member C3 |
| C | 6 | 16593 | ***Klc1*** | kinesin light chain 1 |
| C | 7 | 16551 | ***Kif11*** | kinesin family member 11 |
| C | 8 | 16580 | ***Kifc1*** | kinesin family member C1 |
| C | 9 | 110033 | ***Kif22*** | kinesin family member 22 |
| C | 10 | 17274 | ***Rab8a*** | RAB8A, member RAS oncogene family |
| C | 11 | 17912 | ***Myo1b*** | myosin IB |
| D | 2 | 17879 | ***Myh1*** | myosin, heavy polypeptide 1, skeletal muscle, adult |
| D | 3 | 17882 | ***Myh2*** | myosin, heavy polypeptide 2, skeletal muscle, adult |
| D | 4 | 17883 | ***Myh3*** | myosin, heavy polypeptide 3, skeletal muscle, embryonic |
| D | 5 | 17884 | ***Myh4*** | myosin, heavy polypeptide 4, skeletal muscle |
| D | 6 | 17888 | ***Myh6*** | myosin, heavy polypeptide 6, cardiac muscle, alpha |
| D | 7 | 140781 | ***Myh7*** | myosin, heavy polypeptide 7, cardiac muscle, beta |
| D | 8 | 17885 | ***Myh8*** | myosin, heavy polypeptide 8, skeletal muscle, perinatal |
| D | 9 | 17886 | ***Myh9*** | myosin, heavy polypeptide 9, non-muscle |
| D | 10 | 77579 | ***Myh10*** | myosin, heavy polypeptide 10, non-muscle |
| D | 11 | 546101 | ***EG546101*** | predicted gene, EG546101 |
| E | 2 | 107589 | ***Mylk*** | myosin, light polypeptide kinase |
| E | 3 | 432516 | ***Myo1a*** | myosin IA |
| E | 4 | 17913 | ***Myo1c*** | myosin IC |
| E | 5 | 338367 | ***Myo1d*** | myosin ID |
| E | 6 | 71602 | ***Myo1e*** | myosin IE |
| E | 7 | 17918 | ***Myo5a*** | myosin Va |
| E | 8 | 17919 | ***Myo5b*** | myosin Vb |
| E | 9 | 17920 | ***Myo6*** | myosin VI |
| E | 10 | 17921 | ***Myo7a*** | myosin VIIa |
| E | 11 | 17922 | ***Myo7b*** | myosin VIIb |
| F | 2 | 270163 | ***Myo9a*** | myosin IXa |
| F | 3 | 17925 | ***Myo9b*** | myosin IXb |
| F | 4 | 17909 | ***Myo10*** | myosin X |
| F | 5 | 19341 | ***Rab4a*** | RAB4A, member RAS oncogene family |
| F | 6 | 271457 | ***Rab5a*** | RAB5A, member RAS oncogene family |
| F | 7 | 19344 | ***Rab5b*** | RAB5B, member RAS oncogene family |
| F | 8 | 19345 | ***Rab5c*** | RAB5C, member RAS oncogene family |
| F | 9 | 67117 | ***Dynlt3*** | dynein light chain Tctex-type 3 |
| F | 10 | 21648 | ***Dynlt1*** | dynein light chain Tctex-type 1 |
| F | 11 | 19349 | ***Rab7*** | RAB7, member RAS oncogene family |
| G | 2 | 56455 | ***Dynll1*** | dynein light chain LC8-type 1 |
| G | 3 | 544791 | ***Myh13*** | myosin, heavy polypeptide 13, skeletal muscle |
| G | 4 | 226422 | ***Rab7l1*** | RAB7, member RAS oncogene family-like 1 |
| G | 5 | 16569 | ***Kif3b*** | kinesin family member 3B |
| G | 6 | 71819 | ***Kif23*** | kinesin family member 23 |
| G | 7 | 381293 | ***Kif14*** | kinesin family member 14 |
| G | 8 | 19348 | ***Kif20a*** | kinesin family member 20A |
| G | 9 | 98932 | ***Myl9*** | myosin, light polypeptide 9, regulatory |
| G | 10 | 69654 | ***Dctn2*** | dynactin 2 |
| G | 11 | 67268 | ***2900073G15Rik*** | RIKEN cDNA 2900073G15 gene |
|  |  |  |  |  |
| **PLATE 2** | | | | |
| **row** | **col** | **geneId** | **symbol** | **description** |
| B | 2 | 22428 | ***Dctn6*** | dynactin 6 |
| B | 3 | 16562 | ***Kif1c*** | kinesin family member 1C |
| B | 4 | 73804 | ***Kif2c*** | kinesin family member 2C |
| B | 5 | 16568 | ***Kif3a*** | kinesin family member 3A |
| B | 6 | 53598 | ***Dctn3*** | dynactin 3 |
| B | 7 | 667772 | ***Myh15*** | myosin, heavy chain 15 |
| B | 8 | 381284 | ***E030010N08Rik*** | RIKEN cDNA E030010N08 gene |
| B | 9 | 244281 | ***Myo16*** | myosin XVI |
| B | 10 | 16565 | ***Kif21b*** | kinesin family member 21B |
| B | 11 | 16561 | ***Kif1b*** | kinesin family member 1B |
| C | 2 | 16554 | ***Kif13b*** | kinesin family member 13B |
| C | 3 | 16571 | ***Kif4*** | kinesin family member 4 |
| C | 4 | 668303 | ***Kif26a*** | kinesin family member 26A |
| C | 5 | 218203 | ***Mylip*** | myosin regulatory light chain interacting protein |
| C | 6 | 235661 | ***Dync1li1*** | dynein cytoplasmic 1 light intermediate chain 1 |
| C | 7 | 67665 | ***Dctn4*** | dynactin 4 |
| C | 8 | 17910 | ***Myo15*** | myosin XV |
| C | 9 | 213575 | ***Dync2li1*** | dynein cytoplasmic 2 light intermediate chain 1 |
| C | 10 | 0 | ***siControl*** |  |
| C | 11 | 667663 | ***Myo3a*** | myosin IIIA |
| D | 2 | 17121 | ***Mxd3*** | Max dimerization protein 3 |
| D | 3 | 269152 | ***Kif26b*** | kinesin family member 26B |
| D | 4 | 109242 | ***Kif24*** | kinesin family member 24 |
| D | 5 | 75050 | ***Kif27*** | kinesin family member 27 |
| D | 6 | 16564 | ***Kif21a*** | kinesin family member 21A |
| D | 7 | 208943 | ***Myo5c*** | myosin VC |
| D | 8 | 209737 | ***Kif15*** | kinesin family member 15 |
| D | 9 | 0 | ***siControl*** |  |
| D | 10 | 16559 | ***Kif17*** | kinesin family member 17 |
| D | 11 | 74018 | ***Als2*** | amyotrophic lateral sclerosis 2 (juvenile) homolog (human) |
| E | 2 | 17898 | ***Myl7*** | myosin, light polypeptide 7, regulatory |
| E | 3 | 16553 | ***Kif13a*** | kinesin family member 13A |
| E | 4 | 246177 | ***Myo1g*** | myosin IG |
| E | 5 | 16578 | ***Kif9*** | kinesin family member 9 |
| E | 6 | 16594 | ***Klc2*** | kinesin light chain 2 |
| E | 7 | 0 |  |  |
| E | 8 | 228421 | ***Kif18a*** | kinesin family member 18A |
| E | 9 | 280408 | ***Rilp*** | Rab interacting lysosomal protein |
| E | 10 | 75465 | ***Dynlrb2*** | dynein light chain roadblock-type 2 |
| E | 11 | 67068 | ***Dynlrb1*** | dynein light chain roadblock-type 1 |
| F | 2 | 59288 | ***Dctn5*** | dynactin 5 |
| F | 3 | 73470 | ***Kif2b*** | kinesin family member 2B |
| F | 4 | 74376 | ***Myo18b*** | myosin XVIIIb |
| F | 5 | 228785 | ***Mylk2*** | myosin, light polypeptide kinase 2, skeletal muscle |
| F | 6 | 74764 | ***Klc4*** | kinesin light chain 4 |
| F | 7 | 16581 | ***Kifc2*** | kinesin family member C2 |
| F | 8 | 59310 | ***Mylc2pl*** | myosin light chain 2, precursor lymphocyte-specific |
| F | 9 | 67938 | ***Mylc2b*** | myosin light chain, regulatory B |
| F | 10 | 16552 | ***Kif12*** | kinesin family member 12 |
| F | 11 | 64291 | ***Osbpl1a*** | oxysterol binding protein-like 1A |
| G | 2 | 231836 | ***EG231836*** | predicted gene, EG231836 |
| G | 3 | 216459 | ***Myl6b*** | myosin, light polypeptide 6B |
| G | 4 | 329421 | ***Myo3b*** | myosin IIIB |
| G | 5 | 68097 | ***Dynll2*** | dynein light chain LC8-type 2 |
| G | 6 | 77505 | ***Dnhd1*** | dynein heavy chain domain 1 |
| G | 7 | 327954 | ***Dnahc2*** | dynein, axonemal, heavy chain 2 |
| G | 8 | 232943 | ***Klc3*** | kinesin light chain 3 |
| G | 9 | 319991 | ***Kif6*** | kinesin family member 6 |
| G | 10 | 16576 | ***Kif7*** | kinesin family member 7 |
| G | 11 | 360013 | ***Myo18a*** | myosin XVIIIa |
|  |  |  |  |  |
| **PLATE 3** | | | | |
| **row** | **col** | **geneId** | **symbol** | **description** |
| B | 2 | 17897 | ***Myl3*** | myosin, light polypeptide 3 |
| B | 3 | 17916 | ***Myo1f*** | myosin IF |
| B | 4 | 18196 | ***Nsg1*** | neuron specific gene family member 1 |
| B | 5 | 19325 | ***Rab10*** | RAB10, member RAS oncogene family |
| B | 6 | 23790 | ***Coro1c*** | coronin, actin binding protein 1C |
| B | 7 | 53869 | ***Rab11a*** | RAB11a, member RAS oncogene family |
| B | 8 | 67166 | ***Arl8b*** | ADP-ribosylation factor-like 8B |
| B | 9 | 67845 | ***Zfp364*** | zinc finger protein 364 |
| B | 10 | 72993 | ***Appl1*** | adaptor protein, phosphotyrosine interaction, PH domain and leucine zipper containing 1 |
| B | 11 | 216190 | ***Appl2*** | adaptor protein, phosphotyrosine interaction, PH domain and leucine zipper containing 2 |
| C | 2 | 226421 | ***5430435G22Rik*** | RIKEN cDNA 5430435G22 gene |
| C | 3 | 0 | ***siControl*** |  |
| C | 4 | 668940 | ***Myh7b*** | myosin, heavy chain 7B, cardiac muscle, beta |

**B) siRNA pool targeted sequences**

| **PLATE 1** | | | |
| --- | --- | --- | --- |
| **symbol** | **siRNA 1** | **siRNA 2** | **siRNA 3** |
| ***Kif1a*** | AAGCACCACCACTATTGTCAA | ACGGATAACTGTGACACTGTT | CAGATGCGGGTCTGAGTTGAA |
| ***Bicd1*** | CAGGTTGAATACGAAGGCTTA | TAGAGTCATGTTGGACTACTA | ATGGTTTATGTTTATAAAGAT |
| ***Cenpe*** | CTGGGAGGAAAGAACTCTTAA | CAAGGCTACAATGGTACTATA | AAGCATTGGGCTCGTGAATAA |
| ***Dctn1*** | CCGCATCAAGCTACCAGCTCA | CACAGTCGCCTCATCTCCTAA | AAGGCTGAAATCGAAGAGAAA |
| ***Dync1h1*** | ATGGCTGGGTTTAGTAAATAA | CGGCAAAGAATTCACCAGTAA | TGGCTGGGTTTAGTAAATAAA |
| ***Dync1i1*** | ATGGAACATATTTATTTGAAA | CCCAGTGATGCTGGAAGCCAA | CCGGACAATCCGAGTAATTGA |
| ***Dync1i2*** | ATGGTAGTTATCTATAAATAA | TGGGTGTATATTGTTAAGTTA | CAGGTGCTAAGCTGTCATTAA |
| ***Dync1li2*** | TACCATGACTTTAATGTATAA | TACCATGTTGGCAATGTTCTA | CTTGTTCTAAATGATCTTTAA |
| ***Kif2a*** | CGGGATTTACGTGGAGATCAA | AAGGAGTGCATCCGAGCCTTA | CTGCTGGACCATTCCATCTTA |
| ***Kif3c*** | CAGGGTTTCAACGGCACCGTA | ATGGATCACACCAACGAGCAA | AAGAAGCTCTATGCCAAGCTA |
| ***Kif5c*** | CACTGATTTGACTTAATTTAT | CGAAGTCAGTTTCCAAGATAA | CTGTACAGTAGATTTCATTAA |
| ***Kif5b*** | AGGGATCAAGATAATATGCAA | CAGCAAGAAGTAGACCGGATA | CACGAGCTCACGGTTATGCAA |
| ***Foxc1*** | AAGGATAATTTCCTAAGTAGA | CCCGGCACTCTTAGAGCCAAA | TAGGGTGATCTGCCCTGTCAA |
| ***Kifc3*** | CGGGCTCAGATTGCCATGTAT | CCGCACCACCGAGTTCACCAA | CAGAGGTCTGGGCTATATTTA |
| ***Klc1*** | CAGGGATCAGAACAAGTATAA | AGGGATCAGAACAAGTATAAA | CACCGTCACAACCACCTTGAA |
| ***Kif11*** | AAGGAAGAACTTGAGAATTTA | AGCAAAGAACATAATGAATAA | CACAGGAACTTTGCCAGTTAA |
| ***Kifc1*** | CTGCGCGGAGGTTGAAATTCA | GGCGAGTTACGTAGAGATCTA | TGCGCGGAGGTTGAAATTCAA |
| ***Kif22*** | AGCGAGAGTCCTCAAACCAAA | CAGCATTGAACTTCACTGCTA | AAGGGAAGGCTATGAGCTTAA |
| ***Rab8a*** | CGGACTCGATTGAGAAATTGA | CTCGATGGCAAGAGGATTAAA | CTGGCTCTAATCGGATGTTCA |
| ***Myo1b*** | TGGGATTATGTTAATAATAAA | CAGCAGAAACTTATTTATGAA | CTGGAATTAAATTGTTTCTTA |
| ***Myh1*** | CACAGAGCTGTTCAAGATTAA | AGGGAGAATCTGAATAAGCTA | CGGAACTACTGTAACAGTAAA |
| ***Myh2*** | CCGGGTCACTTTCCAGCTTAA | CAGAGCAAAGATGCAGGGAAA | CAGCTTGATGAAAGGCTCAAA |
| ***Myh3*** | CAGCAGGACCCTGGTGGTTAA | CAGCAGCTGGATACAAAGCTA | CACACTTTGTACGCTGTATAA |
| ***Myh4*** | CTGGATCAACTTGAGACGTTA | CAGGACTTGGTGGACAAACTA | CACACTAAAGTCATAAGCGAA |
| ***Myh6*** | CCGGGTGATCTTCCAGCTAAA | CTCGCTGTGGCCAATACACAA | CAAGCTGATGACAAACCTGAA |
| ***Myh7*** | AAAGAAGGACTTTGAGTTAAA | TGGGAAGACTGTCAACACTAA | TACCAAATCCTGTCTAATAAA |
| ***Myh8*** | CCCAAGGAGTCTTATGTGAAA | CTGGCATCAGCTGATATTGAA | CAGGACCTGGTGGACAAATTA |
| ***Myh9*** | CAGGGCTTATCTACACCTATT | TCCAGCAAGAATGGCTTTGAA | CTCGAGAAAGTCCACTCGGAA |
| ***Myh10*** | CACAAGAATAATGCACTTAAA | CAGTACTAAGACAATGTTAAA | AAGACCAGAATTCCAAATTTA |
| ***EG546101*** | GAGAATACAGTTCACTGTCAA | AGCAGCCAAGATGTGACTTCA | TAAGATGAAATTAGTAACTGA |
| ***Mylk*** | CGCAAAGGATTTCATCAGTAA | CACACTGGTGGTGGAGAACAA | CCGCGTGTCAATGTCAGAGAA |
| ***Myo1a*** | CAGGAGGAATACAAGAGAGAA | CCGAGTGGTGAAGCAACTCAA | CGCGGGCAAGGTGACCTACAA |
| ***Myo1c*** | ATCCAGAGAAACACTAAATAA | AAGACTGAAGCAAATCCTAAA | TTGGATTTGGGCAAATAAATA |
| ***Myo1d*** | ATGAAAGTAATTGGATTCAAA | TGGGAAATACATGGATATCAA | AAGAATTCTATGATTGCTTTA |
| ***Myo1e*** | TTGGTAGATTCTATCAATAAA | AACGATATTATTGATATTATT | CCCAAGCTGTACATTTATAAA |
| ***Myo5a*** | CCCTCGTACTGTTACCTTTAA | CAGCCTTGTATCAATCTTATA | AAGGAAGATGTTAACTGGAAA |
| ***Myo5b*** | CCCGCAGTTCTGCATAACTTA | AACCTGGAGTTTCTCAATGAA | TCAAACTGAGATAATATTAAA |
| ***Myo6*** | CAGATAATTTCCGGTATTTAA | AACCGAGATAATGAAATCTTA | CAGCAGGAGATTGACATGAAA |
| ***Myo7a*** | CCCAGTGATGACTAAGATCTA | CAGAGTCATTCTCCTCCAGAA | CCAGGTGTTCTTCATGAAGAA |
| ***Myo7b*** | CTGCTGAATTTCATTAGTCAA | CAGGTCATTCTGGCTAAGGAA | CACGATGGAGCAGGAAGAGTA |
| ***Myo9a*** | AACTAGGTAATCAATATTAAA | CCCGATGATGATGGTCATTCA | CTGAATTTGAATAAAGTTAAA |
| ***Myo9b*** | CAGCATAACTTGAATATTGAA | CACCTCCTTCCTGAAGAGTAA | CAGAGTGAAATACCAGATCAA |
| ***Myo10*** | CAGGACGAAGCCATCAAGATA | CAGCGCTACAAGAGAAACCAA | CCCAGACGAGAAGATATTTAA |
| ***Rab4a*** | TGGAATTTGGCTCAAAGATAA | ATGCGCTTACTAATTGGTTAA | CAGGTCTGTGACGAGAAGCTA |
| ***Rab5a*** | TAGGAATATGAAACCGATAAA | ATGAATATTGATGACATCTTA | CAAGTTTCTAATTCTGAATTA |
| ***Rab5b*** | CAGGAGCGATACCACAGTTTA | CTCGGGCAAAGACATGGGTAA | TATGATATTACTAATCAGGAA |
| ***Rab5c*** | CCCGACTGGAATCTACTCTAA | CTGGTGGTCATGGTAACCAAA | CACCTGCACCTTTCTGTACAA |
| ***Dynlt3*** | CAGGGAGAAATATATGAACAA | CAGAAAGTAATACATCAAATA | TACACTTGAGGTGGAATTAAA |
| ***Dynlt1*** | CTGTGTGATCATGCAGAAGAA | TTAGAATAATAAATTATTCAA | ATGGGAGAACAAGACCATGTA |
| ***Rab7*** | AGGCCGGAGCTTTGACCATAA | TCCGTTGACCCTGCGCTTCAA | TACAGAGTGTTAGAGACTCAA |
| ***Dynll1*** | CTCCCTTAGGTCATTCCTTTA | ACAGCTTACATTTGTATTTAT | AAGGACTGCATCCAAATTCCA |
| ***Myh13*** | CACGAGCCTGATAAATACCAA | AAGGATGAGGAAATCGAACAA | AAGAGGAGATTTCTGACTTAA |
| ***Rab7l1*** | CCCGTGTACTATAGCTAGTAT | TTGGGTGTCGTCTGTGACTTA | TCCGTTGAGAATATTCAATAT |
| ***Kif3b*** | CTCAAGCATCTTATTATAGAA | AACCATGACCATCCTGTTAAA | CTGCAGGATGAAGATGAGATA |
| ***Kif23*** | AAGGCTGAAGACTATGAAGAA | TTGTTTGAATATGATCTTTAA | TACGATCTATGAGGAAGATAA |
| ***Kif14*** | TTCGTGAATGCTGGAAGTAAA | CTGGCTGGAACTGGGAAATAA | CACGATGATTCAGGAAGCCAA |
| ***Kif20a*** | CAGGAAGTTAAAGCTGAACTA | CAGCTAGATGAAACAAGTCAA | CTCCTTTGCCTTGAAGAGTAA |
| ***Myl9*** | AGGGACAGTCTCTTAATACAA | CAGGGTGAGGCAGGTGCTCTA | CACCAAGGTAAAGCCACTGAA |
| ***Dctn2*** | CAGGAATGAGCCAGACGTTTA | CTGGCCGAGTCTAACACTGTA | TCGGATGAAGAGGCTGGGAAA |
| ***2900073G15Rik*** | CACGTACGCCTTTCTCTCTAA | CACAGGATTCTTTAAGCCCTA | CAGAATTGTGACCCTGCATTA |
|  |  |  |  |
| **PLATE 2** | | | |
| **symbol** | **siRNA 1** | **siRNA 2** | **siRNA 3** |
| ***Dctn6*** | AGCCATGCATTTATTATGTAA | ACCATATACCATAACTAAATA | AACGACCGCTATAATTTATAT |
| ***Kif1c*** | CACGGACGAGCAGAAATTGAA | CAGCAGGTGTACCGAGACATA | ACCTCCATCATTAATCCCAAA |
| ***Kif2c*** | AGCCTCGGTACCGGAACTTAA | CGCCAATATAAGCACAGTGAA | TAGCAGAGTCGTGGTTTCCAA |
| ***Kif3a*** | TGGAGAGAACTCCTTAATTAA | ACGAACCTCCAAAGACATTTA | GAGACCGTAATTGATTCTTTA |
| ***Dctn3*** | TCCAGTGACAATAAACTTGAA | AAGGGTATAACAAGACTACAA | CAGGGTCAGGTTGACCTTAGA |
| ***Myh15*** | AAGGAGGAACTGGAAAGTGAA | AGCGGTGATGATGGACAAGTA | AAGGCCTAATATATCCTAGAA |
| ***E030010N08Rik*** | CTGGTTGAACTTAATCCGCAA | CAGGAGGTGAGTGACCTGCAA | AGGAGGAATCCTGTAAATATA |
| ***Myo16*** | CAGAATCAAGATGAATATAAA | AACCTCTTATTTGTAATGAAA | ACGGGTTATCTTCGGAAGAAA |
| ***Kif21b*** | CTGGTTGTAATTTGAGCACAA | ACCCTTGAAGTTAGGACTAAA | TAAGAGCTTGTCATAAATAAA |
| ***Kif1b*** | ACGAGATTTACTGAATCCCAA | TACAATGACATTGGAAAGGAA | CTCCATCTTCATGCTCACTTA |
| ***Kif13b*** | CACGAGAATGTCCATGAGGAA | CAGGCTGGTCTGCATGGTTAA | CAGGATGTGTCCCAAACACTA |
| ***Kif4*** | CAGTACCAGGATAATATTAAA | AAGAATTGGCTTGGAAATGAA | TACGATGAAATACATGGTCAA |
| ***Kif26a*** | ACGGCAGGAATTCTTACCAAA | CAGGCTTATTGACGAACGCAA | CAGCGGCCTCTTTCTTTATAA |
| ***Mylip*** | CTGCAGTATTGTAAACTATAA | CCGCCTTAAACTGAGGGTCAA | ATCGGGTGCAATACTAGCTAA |
| ***Dync1li1*** | TAGACTTAGTTTATAAATACA | CAGCAGGGTGGGATAATGATA | CCGCAAGTTCTGTTTACAGTA |
| ***Dctn4*** | AAGGATAACCCTGTAATGATA | CAGGATGACCCTGACATCGTA | AAGCAATTACATGTCATGAAA |
| ***Myo15*** | CAGAATGACTTTAAATTCAAA | CACCTTCATTCTGGAACTGAA | CAGAAGCATCATCAAGCAATA |
| ***Dync2li1*** | CTGGTCATTATTGGAAGTAAA | CTCGCTGATGTTTACCAGCAA | TTGGCATTCGGTATTGATAAA |
| ***siControl*** | GAGGCTGAAGATATCTACAAA | AGGCCAAACATTAGCAATTGA | AAGTGCGTGCGTGGAGAGAAA |
| ***Myo3a*** | AAGAGTAGTGCTGTAATAATA | CTGCACTATTATAAATGACAA | CAGGAATTCGACTACAAGAAA |
| ***Mxd3*** | CCCGTATATACTGCACAGTAT | CCACATGTTGAAGAGACTAAA | CCCAGGGAATTTCATGTAGCA |
| ***Kif26b*** | ACCATTCGAAATTAAAGTCTA | CACCAACAAGATGACCCAGAA | TTGCAGCTGAATAAATGTTAA |
| ***Kif24*** | CCGGGTGAAGGAACTAAAGAA | CCCAGACTGATTAGCCAACAA | CAGAGAATCCTTGGACTGAAA |
| ***Kif27*** | CAGGATGAAGTTTATAACACA | CACAATGTGGATCACCTGAAA | CCAGCTGAAGAGAGAACTTAA |
| ***Kif21a*** | AACAAGGTGATGGTCAATCAA | CAGCGCAAGCGAGATCATCAA | CCGTGTCGACATCTTATATTA |
| ***Myo5c*** | ATAGAACTTTATAATACTTAA | CAGCTGATGCTGGATACCAAA | AAGAATGTGCAGAGTAGCTTA |
| ***Kif15*** | AGGCTGGATAATGATATATTA | CAGGATTCCTACGATAACTTA | CACAATAGAATCAATGGAGAA |
| ***siControl*** | TGCGCTTATATCAGCATCTAA | ATCACAGAGTGTGATAAATTA | ATGGGAGCTGATGTTGATCAA |
| ***Kif17*** | TACCATCAACATCGAGATCTA | TCCGCAGAGACTGTAACTATA | CAGGAAGAGATCAAGAGGCTA |
| ***Als2*** | TCCGAGCTACTTTATAATCAA | ACCGATGGACTCAAAGAAGAA | TGCCATTGATTTCCTAAATAA |
| ***Myl7*** | CAGGGATGGGATCATCTGCAA | CAGGCTGAGGTCACCTCAATA | AACTCTGTGCCTGAACTAGAA |
| ***Kif13a*** | AAGGATCTACCTGATCGTGAA | CAGGAGCAGATGAGAAGCATA | CAGGAGGAGGGCTCTAACAAA |
| ***Myo1g*** | CAAGGCTATGATGATCTTGTA | TCCCATCTGTTTGCTGAGCAA | CTCGGTGTGATGGCAAGGATA |
| ***Kif9*** | CAGAAGCACAATTATATGAAA | CAGGAGATTGATGAGACCAAA | ATGGATATAGAAGGTAACCTA |
| ***Klc2*** | AAAGACGATTTGAATCTGAAA | CCGGAGAGCCCTGGAGATCTA | CAGCTGGTACAAAGCCTGTAA |
| ***siControl*** | AAGGACCATTACCGAAAGCTA | CAGGGTCGAGTGGCACAGCTA | AAGCTGAGGCTCAAATATGAA |
| ***Kif18a*** | TTCAAAGATATTGAACATTTA | TGCATTGTAAATATTGTTTAA | CAGATTTATTTGCGACAACAA |
| ***Rilp*** | CAGGAGCGGAATGAGCTCAAA | AGCCATGAAGGTGGCAGTCAA | GAGGAGGAAGATCAAGGCCAA |
| ***Dynlrb2*** | TAGGATCAGATCGAAGAAACA | CCAGCTGACCATGAAAGCCAA | ACGATGGTGGTCAATGCAGAA |
| ***Dynlrb1*** | CTGTGTCATTCCTTAATTTAA | CCCAAGAATAATAGTGCTAAT | ATCCAGAATCCAACTGAATAA |
| ***Dctn5*** | CAAGACCATTATAATGAATGA | AAGCCAGAACATCGTCCTCAA | TAAGACGAACCTGGAGATAAA |
| ***Kif2b*** | AGGGATGAAGTTATTCAGATA | TTGGAGAATTTACATCTTAAA | AAGGCCCAAGATTGTTCTAAA |
| ***Myo18b*** | CAGCAAGACGTCAATCAGCAA | CACCGGGACTTTGACGTAGAA | CTGGTCTTCCAGAATCGACAA |
| ***Mylk2*** | ACCGGAAATGTCAACAGTGAA | CGGGATCCTCTTCATGCACAA | CGCGCCAGATCCAAAGAACAA |
| ***Klc4*** | CACAGTGAACACTACTCTGAA | TCGCTGAACTACTTAAACCAA | CACACCATTGAGTGTCTGCAA |
| ***Kifc2*** | CCGGATCAATTCACATCAGAA | CAGGCATTTGAGAGAGGGCAA | CAGTGTCTGCATCTTCACTTA |
| ***Mylc2pl*** | AAGGCTGACTTCATTAAGGAA | CCAGATGTCTGTGGCAACTTA | TCGCATAAATGTCAAGAACGA |
| ***Mylc2b*** | CAGCATCAGGTCAGATTTAAA | AATGTTGTAAATTGTACTGAA | CACTGTGAAAGCCCAGGACAA |
| ***Kif12*** | AAGGGCCAGAAGGATCAGAAA | AAGGCATATTAGAAAGGGAAA | AAGGCCTCTCTTCTTGTTAAA |
| ***Osbpl1a*** | CACAATGCCAGTGATATTTAA | CTGGGAGAGACTTATGAATTA | CAGGTGACTGATGATGAAGAA |
| ***EG231836*** | CACGGAAGTATCCACCTCAAA | CAGGGAAATTGGGCACCTCAA | CAGCGCCATGTGGCAGAGCAA |
| ***Myl6b*** | CAAAGTGGTGATCGAGTTTAA | AAGGATGCTCCCGTGAAGAAA | CAGCAGGGCCCTCCATCTCTA |
| ***Myo3b*** | CTCGGAGGAAATTGAAGCAAA | AACCACCTTTCTCACAGATAA | TTCCTATTTCATGACATTATA |
| ***Dynll2*** | TAGGTCTGAGGTGGAAGTTAA | CTGGAACAGGTCTGAAGTATA | TAGTTAGGTCTTAGCAATCAA |
| ***Dnhd1*** | CAGGCTCACCTTCATTATTTA | CTGCAGGACCTTAATACTCAA | CCGGTCCTTCATGTACAATTA |
| ***Dnahc2*** | AACCGGGAGCTGAACAAGAAA | ATCGAGGACATCTTTCATAAA | CTCTGGAAATTTATTGACAAA |
| ***Klc3*** | CCCAAATGTGGCCAAGACTAA | ACGGAGGCTAGCCCAAGAGAA | GACCAGAACAAATACAAGGAA |
| ***Kif6*** | CAGGAGGATGATAGCCCATTA | CTGATTCTTCTTAAAGACTAA | AACGGCCTTTATGCACCTGAA |
| ***Kif7*** | ATGGGAGAACTGGGAAAGTAA | TCCCTGCATGAAGATGAACAA | ACAGCTTTCAATGGTGTTATA |
| ***Myo18a*** | CCGGCTGATGCCAAGACGGAA | GACCTGATTGATGTAAGGAAA | ACCCACCATGTTTAACCTCAT |
|  |  |  |  |
| **PLATE 3** | | | |
| **symbol** | **siRNA 1** | **siRNA 2** | **siRNA 3** |
| ***Myl3*** | ACCCTGTGGCCTTATGAATAA | AAGATCGAGTTCACACCTGAA | ACAGAAGATGAGGTAGAGAAA |
| ***Myo1f*** | CACGTCAAAGACATAATCCTA | CAGCCGTAAGATGGACAGCAA | TACCAAGGTCTTTGTTAAGAA |
| ***Nsg1*** | CTGAATCTTGACATTAAAGTA | AGGGACATTTCATTAAATATA | TAGGTCAGAAATAAATGGCAA |
| ***Rab10*** | CTGCACTTCAGTTGTATTATA | CACCATCACAACCTCCTACTA | CTGGATTTGACTAGTCTCCAT |
| ***Coro1c*** | TCCGTTGAATTAATTACGTAA | CTGTGTATGTTTATAAACTAA | CAGAGGGAACTCGGTGATTAT |
| ***Rab11a*** | AAGGCTGTGTATAGTCCATTT | CAGATCTAAGACAACCTTAGA | TAGGACAGAATAGAACTCTAT |
| ***Arl8b*** | AAGAACATTCTGGACAGATAA | TGCCTGCTTTATCTAATGTAA | CCGGTCAATTCAGTGAAGATA |
| ***Zfp364*** | AACCCTCAACCCATAATATTA | CCCAAACTACCGGAATATATA | CCAGCTATCGAAGGAATAATA |
| ***Appl1*** | TAGGAACAAGATAACGATATA | CTGCTATATATTTGAATCAAA | CAGTTAATAATTATCAATTTA |
| ***Appl2*** | CCCATTCAGTTTGATATTGTA | CTGGATTTACAATTTGTTAAA | TGGGAGGGAGACAATAAGAAA |
| ***5430435G22Rik*** | CTCAATGGTATCAACATTCTA | CCGAAAGAAAGTGGACTTGAA | TTCGATGTACATTGCACTGAA |
| ***siControl*** | CTGCGGCAGTTGTTTATGATA | CTGTTTCTTGAAGATGATCTA | AGCAGTGTGAATATGGCTTGA |
| ***Myh7b*** | CTGCGACATAGCCACGAGGAA | CTGGAGTACCAGCGCATGCTA | TCCGATGGCTGGTTTCTCGAA |

**C) H_C_T Z-scores**

| **Plate** | **Well** | **Z-score** | **Well annotation** |
| --- | --- | --- | --- |
| 1 | B03 | 2.05 | sample |
| 3 | B02 | 2.05 | sample |
| 3 | B06 | 1.87 | sample |
| 1 | F09 | 1.71 | sample |
| 2 | G02 | 1.62 | sample |
| 1 | B04 | 1.56 | sample |
| 2 | B09 | 1.56 | sample |
| 3 | B05 | 1.54 | sample |
| 1 | B09 | 1.52 | sample |
| 2 | C03 | 1.38 | sample |
| 1 | F07 | 1.3 | sample |
| 2 | E02 | 1.29 | sample |
| 2 | B10 | 1.28 | sample |
| 1 | G08 | 1.24 | sample |
| 1 | G11 | 1.21 | sample |
| 2 | B02 | 1.14 | sample |
| 1 | E03 | 1.06 | sample |
| 1 | B02 | 1.02 | sample |
| 3 | B07 | 0.93 | sample |
| 1 | F10 | 0.93 | sample |
| 3 | C04 | 0.91 | sample |
| 1 | B08 | 0.88 | sample |
| 2 | E09 | 0.87 | sample |
| 1 | C10 | 0.87 | sample |
| 1 | E10 | 0.86 | sample |
| 2 | D04 | 0.86 | sample |
| 2 | C06 | 0.81 | sample |
| 1 | B06 | 0.74 | sample |
| 1 | B07 | 0.72 | sample |
| 2 | C05 | 0.72 | sample |
| 1 | G04 | 0.68 | sample |
| 2 | B03 | 0.68 | sample |
| 2 | E05 | 0.66 | sample |
| 2 | C01 | 0.64 | siControl |
| 2 | C04 | 0.63 | sample |
| 2 | D10 | 0.61 | sample |
| 1 | C06 | 0.57 | sample |
| 2 | F02 | 0.56 | sample |
| 2 | E04 | 0.51 | sample |
| 1 | G05 | 0.5 | sample |
| 1 | G03 | 0.49 | sample |
| 1 | G09 | 0.48 | sample |
| 2 | F03 | 0.48 | sample |
| 2 | E03 | 0.45 | sample |
| 2 | D11 | 0.42 | sample |
| 1 | F05 | 0.39 | sample |
| 2 | E08 | 0.36 | sample |
| 1 | E04 | 0.34 | sample |
| 3 | B03 | 0.32 | sample |
| 2 | E10 | 0.31 | sample |
| 2 | B08 | 0.29 | sample |
| 1 | E09 | 0.28 | sample |
| 1 | D06 | 0.27 | sample |
| 1 | C01 | 0.23 | siControl |
| 2 | D09 | 0.22 | negative |
| 1 | G07 | 0.22 | sample |
| 2 | B04 | 0.15 | sample |
| 2 | G07 | 0.14 | sample |
| 1 | F04 | 0.13 | sample |
| 1 | E07 | 0.1 | sample |
| 3 | C03 | 0.07 | siControl |
| 2 | F05 | 0.07 | sample |
| 2 | B06 | 0.07 | sample |
| 2 | B07 | 0.07 | sample |
| 1 | E06 | 0.03 | sample |
| 1 | D10 | 0.02 | sample |
| 1 | F02 | 0.01 | sample |
| 2 | F09 | 0.01 | sample |
| 2 | G08 | 0 | sample |
| 1 | D04 | -0.03 | sample |
| 2 | D06 | -0.03 | sample |
| 2 | G03 | -0.05 | sample |
| 2 | D05 | -0.07 | sample |
| 2 | F04 | -0.07 | sample |
| 1 | C11 | -0.07 | sample |
| 1 | D07 | -0.09 | sample |
| 1 | C09 | -0.1 | sample |
| 1 | G02 | -0.1 | sample |
| 1 | C02 | -0.13 | sample |
| 2 | C08 | -0.15 | sample |
| 2 | E11 | -0.18 | sample |
| 2 | B05 | -0.2 | sample |
| 2 | B11 | -0.23 | sample |
| 1 | B10 | -0.26 | sample |
| 1 | E11 | -0.29 | sample |
| 2 | C07 | -0.3 | sample |
| 1 | E08 | -0.31 | sample |
| 3 | B09 | -0.32 | sample |
| 2 | F07 | -0.33 | sample |
| 1 | E05 | -0.34 | sample |
| 2 | D07 | -0.35 | sample |
| 2 | D02 | -0.39 | sample |
| 2 | G04 | -0.46 | sample |
| 1 | C04 | -0.46 | sample |
| 1 | C08 | -0.46 | sample |
| 3 | C02 | -0.48 | sample |
| 3 | B11 | -0.48 | sample |
| 2 | E06 | -0.53 | sample |
| 2 | F06 | -0.53 | sample |
| 1 | F11 | -0.54 | sample |
| 1 | D09 | -0.55 | sample |
| 2 | C11 | -0.55 | sample |
| 1 | C07 | -0.56 | sample |
| 1 | F08 | -0.57 | sample |
| 2 | D03 | -0.57 | sample |
| 1 | D05 | -0.58 | sample |
| 1 | E02 | -0.59 | sample |
| 1 | G10 | -0.6 | sample |
| 3 | B10 | -0.65 | sample |
| 2 | G09 | -0.67 | sample |
| 1 | B11 | -0.68 | sample |
| 3 | B08 | -0.7 | sample |
| 1 | D08 | -0.71 | sample |
| 3 | B04 | -0.71 | sample |
| 2 | G11 | -0.71 | sample |
| 2 | C10 | -0.77 | siControl |
| 1 | D11 | -0.79 | sample |
| 1 | G06 | -0.82 | sample |
| 1 | C03 | -0.86 | sample |
| 2 | F08 | -0.87 | sample |
| 1 | F03 | -0.91 | sample |
| 1 | F06 | -0.95 | sample |
| 2 | G06 | -0.96 | sample |
| 2 | D08 | -0.99 | sample |
| 1 | D02 | -1.04 | sample |
| 1 | C05 | -1.06 | sample |
| 2 | C09 | -1.09 | sample |
| 1 | D03 | -1.09 | sample |
| 2 | F11 | -1.18 | sample |
| 2 | F10 | -1.19 | sample |
| 2 | G05 | -1.23 | sample |
| 3 | C07 | -1.34 | siControl |
| 2 | E07 | -1.37 | siControl |
| 1 | B05 | -1.45 | sample |
| 2 | F12 | -1.53 | EHNA |
| 2 | G10 | -1.64 | sample |
| 2 | C02 | -1.72 | sample |
| 2 | C12 | -1.76 | EHNA |
| 3 | D03 | -1.91 | EHNA |
| 1 | F12 | -2.14 | EHNA |
| 1 | C12 | -2.71 | EHNA |
| 3 | D06 | -2.74 | EHNA |

**D) p75^NTR^ Z-scores**

| **Plate** | **Well** | **Z-score** | **Well annotation** |
| --- | --- | --- | --- |
| 1 | G03 | 4.06 | sample |
| 1 | G04 | 4.06 | sample |
| 1 | G10 | 3.66 | sample |
| 3 | B02 | 3.17 | sample |
| 1 | G02 | 3.15 | sample |
| 1 | F11 | 2.92 | sample |
| 1 | F03 | 2.61 | sample |
| 2 | G02 | 2.43 | sample |
| 3 | C04 | 2.18 | sample |
| 1 | F04 | 2.12 | sample |
| 2 | E06 | 2.09 | sample |
| 2 | F09 | 1.93 | sample |
| 2 | C06 | 1.84 | sample |
| 2 | D09 | 1.74 | sample |
| 2 | B06 | 1.71 | sample |
| 2 | E02 | 1.71 | sample |
| 3 | B05 | 1.54 | sample |
| 1 | G05 | 1.44 | sample |
| 2 | C09 | 1.38 | sample |
| 2 | F02 | 1.15 | sample |
| 1 | B06 | 1.1 | sample |
| 2 | G03 | 0.86 | sample |
| 1 | E02 | 0.86 | sample |
| 1 | B08 | 0.74 | sample |
| 1 | C03 | 0.74 | sample |
| 2 | F05 | 0.74 | sample |
| 1 | D02 | 0.71 | sample |
| 1 | E03 | 0.71 | sample |
| 1 | G07 | 0.68 | sample |
| 2 | F04 | 0.67 | sample |
| 2 | G07 | 0.65 | sample |
| 1 | D06 | 0.61 | sample |
| 1 | D03 | 0.58 | sample |
| 1 | G08 | 0.58 | sample |
| 1 | F08 | 0.57 | sample |
| 1 | B03 | 0.57 | sample |
| 2 | C01 | 0.52 | siControl |
| 2 | E04 | 0.5 | sample |
| 1 | E10 | 0.49 | sample |
| 2 | F08 | 0.45 | sample |
| 2 | C05 | 0.44 | sample |
| 2 | B03 | 0.43 | sample |
| 2 | B09 | 0.43 | sample |
| 2 | C02 | 0.42 | sample |
| 3 | B07 | 0.42 | sample |
| 2 | C07 | 0.41 | sample |
| 2 | F06 | 0.4 | sample |
| 2 | D05 | 0.39 | sample |
| 2 | E08 | 0.38 | sample |
| 1 | D05 | 0.37 | sample |
| 1 | E05 | 0.34 | sample |
| 1 | F02 | 0.31 | sample |
| 1 | G09 | 0.27 | sample |
| 2 | B04 | 0.25 | sample |
| 1 | F10 | 0.19 | sample |
| 1 | B09 | 0.19 | sample |
| 2 | D04 | 0.14 | sample |
| 1 | E11 | 0.14 | sample |
| 3 | B09 | 0.13 | sample |
| 2 | B07 | 0.13 | sample |
| 1 | B04 | 0.12 | sample |
| 2 | E11 | 0.12 | sample |
| 1 | F09 | 0.11 | sample |
| 1 | E09 | 0.09 | sample |
| 2 | G04 | 0.09 | sample |
| 1 | B05 | 0.06 | sample |
| 2 | E03 | 0.06 | sample |
| 3 | B11 | 0.06 | sample |
| 2 | C10 | 0.04 | siControl |
| 1 | E08 | 0 | sample |
| 2 | G10 | 0 | sample |
| 2 | B08 | -0.03 | sample |
| 2 | F03 | -0.04 | sample |
| 1 | D04 | -0.1 | sample |
| 1 | C04 | -0.11 | sample |
| 1 | G06 | -0.11 | sample |
| 3 | B04 | -0.13 | sample |
| 3 | C07 | -0.17 | siControl |
| 2 | E05 | -0.17 | sample |
| 2 | B02 | -0.19 | sample |
| 2 | D07 | -0.2 | siControl |
| 2 | C11 | -0.23 | sample |
| 1 | F05 | -0.25 | sample |
| 2 | C04 | -0.25 | sample |
| 2 | E07 | -0.25 | siControl |
| 1 | F07 | -0.26 | sample |
| 3 | B03 | -0.27 | sample |
| 2 | G05 | -0.28 | sample |
| 2 | C08 | -0.28 | sample |
| 2 | D06 | -0.29 | sample |
| 2 | E09 | -0.3 | sample |
| 3 | B10 | -0.31 | sample |
| 1 | C02 | -0.32 | sample |
| 1 | C01 | -0.33 | siControl |
| 1 | F06 | -0.36 | sample |
| 1 | D09 | -0.38 | sample |
| 1 | D10 | -0.4 | sample |
| 1 | E06 | -0.41 | sample |
| 2 | C03 | -0.42 | sample |
| 2 | G11 | -0.42 | sample |
| 3 | B06 | -0.45 | sample |
| 1 | D08 | -0.49 | sample |
| 1 | C08 | -0.53 | sample |
| 2 | G08 | -0.54 | sample |
| 1 | G11 | -0.57 | sample |
| 1 | C06 | -0.58 | sample |
| 1 | D07 | -0.58 | sample |
| 2 | G06 | -0.59 | sample |
| 1 | B11 | -0.6 | sample |
| 2 | B11 | -0.67 | sample |
| 1 | E07 | -0.71 | sample |
| 3 | C02 | -0.74 | sample |
| 1 | E04 | -0.76 | sample |
| 2 | D10 | -0.78 | sample |
| 1 | D11 | -0.78 | sample |
| 2 | D11 | -0.79 | sample |
| 3 | B08 | -0.8 | sample |
| 2 | F07 | -0.86 | sample |
| 1 | C09 | -0.87 | sample |
| 3 | C03 | -0.89 | siControl |
| 1 | C05 | -0.9 | sample |
| 2 | G09 | -0.9 | sample |
| 1 | C07 | -0.97 | sample |
| 2 | B05 | -0.97 | sample |
| 2 | F10 | -0.98 | sample |
| 1 | C10 | -1.02 | sample |
| 1 | B02 | -1.11 | sample |
| 2 | F11 | -1.15 | sample |
| 2 | D08 | -1.18 | sample |
| 1 | C11 | -1.22 | sample |
| 2 | D02 | -1.23 | sample |
| 1 | B10 | -1.26 | sample |
| 2 | E10 | -1.36 | sample |
| 2 | D03 | -1.36 | sample |
| 1 | B07 | -1.47 | sample |
| 2 | B10 | -1.87 | sample |
| 2 | F12 | -2.27 | EHNA |
| 2 | C12 | -2.36 | EHNA |
| 1 | F12 | -2.5 | EHNA |
| 3 | D02 | -2.58 | EHNA |
| 1 | C12 | -2.77 | EHNA |

**E) Plate layout**

| **Well** | **Gene Symbol** | **Gene Id** | **Accession Number** | **Sequence** |
| --- | --- | --- | --- | --- |
| B03 | ***Myh13*** | 544791 | NM_001081250 | GCGCAAAGUUAAAGAGAUG |
| B03 | ***Myh13*** | 544791 | NM_001081250 | AAUCUGAGCUUGAUCGCAA |
| B03 | ***Myh13*** | 544791 | NM_001081250 | GGAUGAGGAAAUCGAACAA |
| B03 | ***Myh13*** | 544791 | NM_001081250 | GAUAAAGAAAUGUACGUGA |
| B04 | ***Rab7l1*** | 226422 | NM_144875 | UGACACGACUCUACUAUAG |
| B04 | ***Rab7l1*** | 226422 | NM_144875 | CAAGGGAACUACAUCAAUC |
| B04 | ***Rab7l1*** | 226422 | NM_144875 | GGACAGCAAGCUCACACUA |
| B04 | ***Rab7l1*** | 226422 | NM_144875 | GCCGAGAUCACCUGUUUAA |
| B05 | ***Dctn2*** | 69654 | NM_027151 | GCAGAAGUACCAACGACUA |
| B05 | ***Dctn2*** | 69654 | NM_027151 | GAACUGGUACAGCGACUUG |
| B05 | ***Dctn2*** | 69654 | NM_027151 | GGAGGCCACUGUCCGAUGU |
| B05 | ***Dctn2*** | 69654 | NM_027151 | GAGCCAGACGUUUAUGAAA |
| B06 | ***Myl3*** | 17897 | NM_010859 | UUUGAUGCCUCCAAGAUUA |
| B06 | ***Myl3*** | 17897 | NM_010859 | CAAGGACACUGGCACGUAC |
| B06 | ***Myl3*** | 17897 | NM_010859 | GCGAGAUGAAGAUCACAUA |
| B06 | ***Myl3*** | 17897 | NM_010859 | GGUGAGAGACUGACAGAAG |
| B07 | ***Dynll1*** | 56455 | NM_019682 | GACAAGAAGUACAACCCUA |
| B07 | ***Dynll1*** | 56455 | NM_019682 | GCAGACAUGUCGGAAGAGA |
| B07 | ***Dynll1*** | 56455 | NM_019682 | UACAACAUCGAGAAGGAUA |
| B07 | ***Dynll1*** | 56455 | NM_019682 | CUUCGGUAGUUAUGUGGCA |
| B08 | ***Rab7*** | 19349 | NM_009005 | GUACAAAGCCACAAUAGGA |
| B08 | ***Rab7*** | 19349 | NM_009005 | AAACAACAUUCCUUACUUC |
| B08 | ***Rab7*** | 19349 | NM_009005 | AAACAAGAUUGACCUGGAA |
| B08 | ***Rab7*** | 19349 | NM_009005 | AAGUGGAACUGUACAAUGA |
| B09 | ***Myo9b*** | 17925 | NM_015742 | CAACCGGACACGGGAAUUA |
| B09 | ***Myo9b*** | 17925 | NM_015742 | GAACCGAAAUCGCAAAGUU |
| B09 | ***Myo9b*** | 17925 | NM_015742 | CCAAUGAGCUCAAGUUUCU |
| B09 | ***Myo9b*** | 17925 | NM_015742 | CAGGAAGACUCUAGACGUA |
| B10 | ***EG231836*** | 231836 | XM_144611 | GAGCGGACCUUUAUAUUUG |
| B10 | ***EG231836*** | 231836 | XM_144611 | GGAUACAACACGACCAUUU |
| B10 | ***EG231836*** | 231836 | XM_144611 | UGAGGGAGAUGGAACCCAA |
| B10 | ***EG231836*** | 231836 | XM_144611 | GCAGGUGACCAGUGAGGAA |
| B11 | ***Myh7b*** | 668940 | NM_001085378 | GGACAGAGCUCUUUCGACU |
| B11 | ***Myh7b*** | 668940 | NM_001085378 | ACAAAUGGCUCCCGGUCUA |
| B11 | ***Myh7b*** | 668940 | NM_001085378 | CACAAUUUCUGGCGACAAA |
| B11 | ***Myh7b*** | 668940 | NM_001085378 | GCAUGGAGGUGGACGAUCU |
| C03 | ***Myo10*** | 17909 | NM_019472 | ACAAUUCCCUGGACUACUA |
| C03 | ***Myo10*** | 17909 | NM_019472 | GCAAUGCGAAGACAGUAUA |
| C03 | ***Myo10*** | 17909 | NM_019472 | GUACAUGAAAGGUGGCUUA |
| C03 | ***Myo10*** | 17909 | NM_019472 | AGACCCAACUCAUUUGUGA |
| C04 | ***Klc2*** | 16594 | NM_008451 | GCGAGGAGAGCAAGGAUAA |
| C04 | ***Klc2*** | 16594 | NM_008451 | GGACGGGUCUUUCUGACAG |
| C04 | ***Klc2*** | 16594 | NM_008451 | CAAUGAAGAUGAACAGAGC |
| C04 | ***Klc2*** | 16594 | NM_008451 | CCACAGGAGCCUAACUCUA |
| C05 | ***Mylc2b*** | 67938 | NM_023402 | GCGCGAAAGACAAAGAUGA |
| C05 | ***Mylc2b*** | 67938 | NM_023402 | GCGCGCAACCUCCAAUGUG |
| C05 | ***Mylc2b*** | 67938 | NM_023402 | GGGAACUUCAACUACAUUG |
| C05 | ***Mylc2b*** | 67938 | NM_023402 | UCGCUUGCUUUGAUGAGGA |
| C06 | ***Rab10*** | 19325 | NM_016676 | GGAAUAGACUUUAAGAUCA |
| C06 | ***Rab10*** | 19325 | NM_016676 | CAAGAGAGUUGUACCGAAA |
| C06 | ***Rab10*** | 19325 | NM_016676 | CACAUUAGCUGAAGACAUC |
| C06 | ***Rab10*** | 19325 | NM_016676 | UAGAUGAGCAUGCCAAUGA |
| C07 | ***Bicd1*** | 12121 | NM_009753 | GAUGAAAUCCGAGAAUAUA |
| C07 | ***Bicd1*** | 12121 | NM_009753 | CCAAAUGUAUGAUGAACAA |
| C07 | ***Bicd1*** | 12121 | NM_009753 | GAAGUGAGCCAAAUAACGA |
| C07 | ***Bicd1*** | 12121 | NM_009753 | UUACAAAUGUCCAGGCAGA |
| C08 | ***Coro1c*** | 23790 | NM_011779 | GAUCAAAUCCAUAAAGGAA |
| C08 | ***Coro1c*** | 23790 | NM_011779 | GACCGAGCCUGUGGUGAUU |
| C08 | ***Coro1c*** | 23790 | NM_011779 | CAAUUGCUCUCCAUGAAAU |
| C08 | ***Coro1c*** | 23790 | NM_011779 | GAAAGUGCGAGCCCAUUAU |
| C09 | ***Dynlt3*** | 67117 | NM_025975 | CCCAUAAUAUAGUCAAAGA |
| C09 | ***Dynlt3*** | 67117 | NM_025975 | GGUGGUAACGAUUAUAAUG |
| C09 | ***Dynlt3*** | 67117 | NM_025975 | GGGGAAAGCUUACAAGUAC |
| C09 | ***Dynlt3*** | 67117 | NM_025975 | CAGAGGAGCCCGUAUGGAU |
| C10 | ***Cenpe*** | 229841 | NM_173762 | GAAGAGCUCCAUAUAAUAA |
| C10 | ***Cenpe*** | 229841 | NM_173762 | GAUUAUGAGUGCUUGAAUA |
| C10 | ***Cenpe*** | 229841 | NM_173762 | GAUAGCAAAUUGACACGAA |
| C10 | ***Cenpe*** | 229841 | NM_173762 | GAACUUAACCUUGCUCGUU |
| C11 | ***Myo16*** | 244281 | XM_356059 | GGAAACAUCCCUUUAGAUU |
| C11 | ***Myo16*** | 244281 | XM_356059 | CAUCACAACUCACGAAAUC |
| C11 | ***Myo16*** | 244281 | XM_356059 | GAGUGUAGAUCUAUGAAUC |
| C11 | ***Myo16*** | 244281 | XM_356059 | GGACUUCACCUUAAUAACU |
| D03 | ***Dync1li2*** | 234663 | NM_001013380 | GAAGAUGGUUCUGGUAAGA |
| D03 | ***Dync1li2*** | 234663 | NM_001013380 | UAAAGAAGCCAGAUCCAAA |
| D03 | ***Dync1li2*** | 234663 | NM_001013380 | GAAAGCUGGUCCAUGACAA |
| D03 | ***Dync1li2*** | 234663 | NM_001013380 | UUACAACCGUGAAGCCAGA |
| D04 | ***Kif21b*** | 16565 | NM_019962 | GGGUAAAGCUGUGGAAUUA |
| D04 | ***Kif21b*** | 16565 | NM_019962 | GAACCAGUCUCGCUAUGAA |
| D04 | ***Kif21b*** | 16565 | NM_019962 | CAACGUGGUUUCUAUCAAA |
| D04 | ***Kif21b*** | 16565 | NM_019962 | CAAACUGACUGGCCACAUU |
| D05 | ***Dync1i1*** | 13426 | NM_010063 | GGAAGGGGCUGUUGAGUUA |
| D05 | ***Dync1i1*** | 13426 | NM_010063 | GCAUUUGGAUCUAUGAUGU |
| D05 | ***Dync1i1*** | 13426 | NM_010063 | CCACAAUGCUCGCAACCUG |
| D05 | ***Dync1i1*** | 13426 | NM_010063 | GACAAUCGCAGUCAUCGAA |
| D06 | ***Kif26b*** | 269152 | NM_177757 | GCGAGGAUCUGGAGUGCUA |
| D06 | ***Kif26b*** | 269152 | NM_177757 | GAAAGCCUGUCGUCGGUGA |
| D06 | ***Kif26b*** | 269152 | NM_177757 | GAUCGCGUCGAGAGUCUUA |
| D06 | ***Kif26b*** | 269152 | NM_177757 | AAGACUAGAUUGCAUAGAU |
| D07 | ***Dynlrb2*** | 75465 | NM_029297 | GAACGAUGGUGGUCAAUGC |
| D07 | ***Dynlrb2*** | 75465 | NM_029297 | GGACAACUCCACAACGGUU |
| D07 | ***Dynlrb2*** | 75465 | NM_029297 | GAGAAUCCAGAGUCACAAA |
| D07 | ***Dynlrb2*** | 75465 | NM_029297 | AGAUCGAAGAAACAUGAAA |
| D08 | ***Kif2a*** | 16563 | NM_008442 | GUAAAGGAGUUUGGAAUUA |
| D08 | ***Kif2a*** | 16563 | NM_008442 | GAAAUGGUUUACAGGUUUA |
| D08 | ***Kif2a*** | 16563 | NM_008442 | UGAAGAAGCCAAACUAUAA |
| D08 | ***Kif2a*** | 16563 | NM_008442 | GGAAUGGCAUCCUGUGAAA |
| D09 | ***Mxd3*** | 17121 | NM_016662 | GGAGUUGACUGUACCCGAU |
| D09 | ***Mxd3*** | 17121 | NM_016662 | GCGCUCAGACUCAGACCAA |
| D09 | ***Mxd3*** | 17121 | NM_016662 | GGGCCAGGGUGCAUAUCCA |
| D09 | ***Mxd3*** | 17121 | NM_016662 | AGGAAAAGCUUCGCAGCAA |
| D10 | ***Myo1b*** | 17912 | NM_010863 | GAGCUUACCUGGAAAUCAA |
| D10 | ***Myo1b*** | 17912 | NM_010863 | GGACUCUGCUAAAGUUAAU |
| D10 | ***Myo1b*** | 17912 | NM_010863 | CAUCUAGAUUCGGCAAAUA |
| D10 | ***Myo1b*** | 17912 | NM_010863 | GAUCGGUGUUGGAGAUAUG |
| D11 | ***Kif15*** | 209737 | NM_010620 | GAACAACUGUCUCAAUUUA |
| D11 | ***Kif15*** | 209737 | NM_010620 | GAACAGACGUGUAGCAUCA |
| D11 | ***Kif15*** | 209737 | NM_010620 | GGACAACGCCAGAUUAGAA |
| D11 | ***Kif15*** | 209737 | NM_010620 | GGAUUCCUACGAUAACUUA |
| E03 | ***Osbpl1a*** | 64291 | NM_020573 | GGGAGAGACUUAUGAAUUA |
| E03 | ***Osbpl1a*** | 64291 | NM_020573 | GCACAGGACUGGAUUUAUU |
| E03 | ***Osbpl1a*** | 64291 | NM_020573 | GCAAGGAAUUACACAAAGU |
| E03 | ***Osbpl1a*** | 64291 | NM_020573 | CAACGAAGCAUACACAUGG |
| E04 | ***Kif1a*** | 16560 | NM_008440 | AGACAGAUUUCAUCCCUUA |
| E04 | ***Kif1a*** | 16560 | NM_008440 | GGAAGCACCACCACUAUUG |
| E04 | ***Kif1a*** | 16560 | NM_008440 | GAAGUGCGCGAGCUAGUUG |
| E04 | ***Kif1a*** | 16560 | NM_008440 | ACACAUAUGUCAACGGCAA |
| E05 | ***Rab8a*** | 17274 | NM_023126 | GAAUAAGUGUGAUGUGAAU |
| E05 | ***Rab8a*** | 17274 | NM_023126 | GAAGACCUGUGUCCUGUUC |
| E05 | ***Rab8a*** | 17274 | NM_023126 | GACCUACGAUUACCUGUUC |
| E05 | ***Rab8a*** | 17274 | NM_023126 | GAGCAGCCAUGGAGUCAAG |
| E06 | ***Kif12*** | 16552 | NM_010616 | CGUCGGAGCUCCUCACAUA |
| E06 | ***Kif12*** | 16552 | NM_010616 | GAACAUGCCAUAUGGGCUA |
| E06 | ***Kif12*** | 16552 | NM_010616 | GAUGUGGCGUUCCGCUUUG |
| E06 | ***Kif12*** | 16552 | NM_010616 | GCUAUUAGAUCGCGUGCAG |
| E07 | ***Kif3a*** | 16568 | NM_008443 | CAUGAUGUGUGCAAAUAUU |
| E07 | ***Kif3a*** | 16568 | NM_008443 | AGACUUAUCAGCAUAUGUA |
| E07 | ***Kif3a*** | 16568 | NM_008443 | CGACUAAUAUGAACGAGCA |
| E07 | ***Kif3a*** | 16568 | NM_008443 | CCUGAGACCGUAAUUGAUU |
| E08 | ***Kif11*** | 16551 | NM_010615 | GUAAAUGGCUGUUGUAAAG |
| E08 | ***Kif11*** | 16551 | NM_010615 | CAGCAGAGGUCUUCCAUUU |
| E08 | ***Kif11*** | 16551 | NM_010615 | GGAGAUCACUAAGAAAGUA |
| E08 | ***Kif11*** | 16551 | NM_010615 | GAAACAGGAUCUGAAACUA |
| E09 | ***Arl8c*** | 67166 | NM_026011 | GAUAGAAGCCUCUCGAAAU |
| E09 | ***Arl8c*** | 67166 | NM_026011 | UAAAGGCAACGUCACAAUA |
| E09 | ***Arl8c*** | 67166 | NM_026011 | GAUAGAUGCUGCAGAUCGA |
| E09 | ***Arl8c*** | 67166 | NM_026011 | UAGAGAAAUUUGCUGCUAU |
| E10 | ***Kif13b*** | 16554 | XM_283218 | GUACUUAGAUGCUGCCUUA |
| E10 | ***Kif13b*** | 16554 | XM_283218 | CAGCCUACCUUCUCAGAUA |
| E10 | ***Kif13b*** | 16554 | XM_283218 | GAACAUGGAUUGGUGUGGA |
| E10 | ***Kif13b*** | 16554 | XM_283218 | GUGCGGACCUCAGAGCUUA |
| E11 | ***Kif7*** | 16576 | XM_133575 | AAAGAGAGCAUCCAGAUUA |
| E11 | ***Kif7*** | 16576 | XM_133575 | GAAUGAGGCUAUCACAUGC |
| E11 | ***Kif7*** | 16576 | XM_133575 | GGUCGACACAUGUGGAUAA |
| E11 | ***Kif7*** | 16576 | XM_133575 | GAUGAUCGACGUCAGGAAA |
| F03 | ***Dctn1*** | 13191 | NM_007835 | GGACAGCGCUGCCAAGGAU |
| F03 | ***Dctn1*** | 13191 | NM_007835 | CAAAGGUCCUCAAGAGAGA |
| F03 | ***Dctn1*** | 13191 | NM_007835 | GAGCAAGCCUCCUCCGGUU |
| F03 | ***Dctn1*** | 13191 | NM_007835 | CACGAGCGCUCCUUAGAUU |
| F04 | ***Dynll2*** | 68097 | NM_026556 | ACAAAGCACUUCAUCUAUU |
| F04 | ***Dynll2*** | 68097 | NM_026556 | GCAGCUAUGUCACACACGA |
| F04 | ***Dynll2*** | 68097 | NM_026556 | AACCCUACCUGGCAUUGUA |
| F04 | ***Dynll2*** | 68097 | NM_026556 | GCCUAUAUCAAGAAGGAAU |
| F05 | ***Myh2*** | 17882 | NM_144961 | CCACGAACCCAUAUGAUUA |
| F05 | ***Myh2*** | 17882 | NM_144961 | GCGGAAAGAAGCUACCAUA |
| F05 | ***Myh2*** | 17882 | NM_144961 | CCGGUGCUGUGAUGCAUUA |
| F05 | ***Myh2*** | 17882 | NM_144961 | GCGCUGGUAUCUCAGUUAU |
| F06 | ***Dync2li1*** | 213575 | NM_172256 | GGACGGAGCUGAAAUCGGA |
| F06 | ***Dync2li1*** | 213575 | NM_172256 | GGCAUUCGGUAUUGAUAAA |
| F06 | ***Dync2li1*** | 213575 | NM_172256 | GUAGAGAAACGGAGAAGUC |
| F06 | ***Dync2li1*** | 213575 | NM_172256 | CAACCAUUAUCCUAAGGUG |
| F07 | ***Kifc3*** | 16582 | NM_010631 | GGAAGUGGCUGAGAACAAA |
| F07 | ***Kifc3*** | 16582 | NM_010631 | GGGCUCAGAUUGCCAUGUA |
| F07 | ***Kifc3*** | 16582 | NM_010631 | CACGUGAGAUUUACAAUGA |
| F07 | ***Kifc3*** | 16582 | NM_010631 | GGAAACAUCCGGGUGAUUG |
| F08 | ***Myh1*** | 17879 | XM_354615 | UGAAAGAACUCACUUAUCA |
| F08 | ***Myh1*** | 17879 | XM_354615 | GAAGCCGGAUCUAAUCGAA |
| F08 | ***Myh1*** | 17879 | XM_354615 | GCAGAGAUAUAAGGUUUUA |
| F08 | ***Myh1*** | 17879 | XM_354615 | GAACAGAAGCGCAACGUGG |
| F09 | ***Dnhd1*** | 77505 | XM_355968 | CAAUAUGUCUGUCGAAUUA |
| F09 | ***Dnhd1*** | 77505 | XM_355968 | GCAUGAGCGUCAAGUAUUU |
| F09 | ***Dnhd1*** | 77505 | XM_355968 | GCAGAUACCUAUGAAGCUA |
| F09 | ***Dnhd1*** | 77505 | XM_355968 | GGAGAAUGAUCACAGUCUA |
| F10 | ***Rab5a*** | 271457 | NM_025887 | AAACAAAGCUGACUUAGCA |
| F10 | ***Rab5a*** | 271457 | NM_025887 | GGUCAAGAACGGUAUCAUA |
| F10 | ***Rab5a*** | 271457 | NM_025887 | GCACAAGCAGCCAUAGUUG |
| F10 | ***Rab5a*** | 271457 | NM_025887 | GGCCAAAUACUGGAAAUAA |

**F) H_C_T validation scores**

| **Mean of the siControls** | **Mean of EHNA treated samples** |  |
| --- | --- | --- |
| **1.1911315** | **0.11340095** |  |
| Increased INTRACELLULAR Accumulation | | |
| **Candidate name** | **Score** | **Score minus the median of the siControls** |
| **BICD1** | 1.831421 | 0.6402895 |
| **MYL3** | 1.2197258 | 0.0285943 |
| **CORO1C** | 1.26823372 | 0.07710222 |
| **Dynlt3** | 0.66562036 | -0.52551114 |
| **EG231836** | 1.24756302 | 0.05643152 |
| **Myo16** | 1.42768216 | 0.23655066 |
| **CENPE** | 0.8220231 | -0.3691084 |
| **RAB10** | 0.95982068 | -0.23131082 |
| **Dync1li2** | 1.1063869 | -0.0847446 |
| Decreased iintracellular accumulation | | |
| **Candidate name** | **Score** | **Score minus the median of the siControls** |
| **KIF13B** | 0.86119454 | -0.32993696 |
| **KIF7** | 0.87835562 | -0.31277588 |
| **DCTN1** | 0.75369896 | -0.43743254 |
| **Dynll2** | 1.0708651 | -0.1202664 |
| **KIF12** | 1.1888181 | -0.0023134 |
| **OSBPL1A** | 0.87861126 | -0.31252024 |
| **MYH2** | 1.05655968 | -0.13457182 |
| **Dync2li1** | 0.78353784 | -0.40759366 |
| **KIF3C** | 0.70126436 | -0.48986714 |
| **MYH1** | 1.01950226 | -0.17162924 |
| **KIF15** | 0.71261168 | -0.47851982 |
| **Dnhd1** | 1.16207428 | -0.02905722 |
| **RAB5A** | 1.2500167 | 0.0588852 |
| **ARL8C** | 1.31220688 | 0.12107538 |

**G) p75^NTR^ validation score**

| **Median of the siControls** | **Median of EHNA treated samples** |  |
| --- | --- | --- |
| **1.313071** | **0.1074658** |  |
| Increased intracellular accumulation | | |
| **Candidate name** | **Score** | **Score minus the median of the siControls** |
| **Myh13** | 1.48087468 | 0.16780368 |
| **RAB7L1** | 0.85880744 | -0.45426356 |
| **DCTN2** | 0.9419114 | -0.3711596 |
| **MYL3** | 1.23503192 | -0.07803908 |
| **Dynll1** | 1.22249072 | -0.09058028 |
| **RAB7** | 1.5793482 | 0.2662772 |
| **MYO9B** | 0.88561824 | -0.42745276 |
| **EG231836** | 0.80367186 | -0.50939914 |
| **Myh7b** | 0.50435852 | -0.80871248 |
| **MYO10** | 0.85313542 | -0.45993558 |
| **MYLC2B** | 1.27796624 | -0.03510476 |
| **RAB10** | 1.15070274 | -0.16236826 |
| Decreased intracellular accumulation | | |
| **Candidate name** | **Score** | **Score minus the median of the siControls** |
| **KIF21B** | 0.73966624 | -0.57340476 |
| **Dync1i1** | 1.16264346 | -0.15042754 |
| **KIF26B** | 0.90418152 | -0.40888948 |
| **Dynlrb2** | 0.95027282 | -0.36279818 |
| **KIF2A** | 1.13100118 | -0.18206982 |
| **MXD3** | 0.73556886 | -0.57750214 |
| **MYO1B** | 1.09169806 | -0.22137294 |
| **KIF15** | 1.0111803 | -0.3018907 |
| **OSBPL1A** | 1.12998484 | -0.18308616 |
| **KIF1A** | 1.05208572 | -0.26098528 |
| **RAB8A** | 1.3917102 | 0.0786392 |
| **KIF12** | 1.32336156 | 0.01029056 |
| **KIF3A** | 1.5711236 | 0.2580526 |
| **KIF11** | 0.84326322 | -0.46980778 |
| **ARL8C** | 1.09350966 | -0.21956134 |
